# Multiscale characterization of the human claustrum from histology to MRI

**DOI:** 10.1073/pnas.2604111123

**Published:** 2026-06-29

**Authors:** Navona Calarco, Skerdi Progri, Sriranga Kashyap, Shuting Xie, Claude Lepage, Donna Gift Cabalo, Boris C. Bernhardt, Alan C. Evans, Kâmil Uludağ

**Affiliations:** ^a^ Krembil Brain Institute, University Health Network, Toronto, ON M5T 2S8, Canada; ^b^ Department of Medical Biophysics, University of Toronto, Toronto, ON M5G 2C4, Canada; ^c^ Physical Sciences Platform, Sunnybrook Research Institute,Toronto, ON M4N 3M5, Canada; ^d^ McConnell Brain Imaging Centre, Montreal Neurological Institute and Hospital, McGill University, Montreal, QC H3A 0G4, Canada; ^e^ Harquail Centre for Neuromodulation, Hurvitz Brain Sciences Program, Sunnybrook Research Institute, Toronto, ON M4N 3M5, Canada; ^f^ Center for Neuroscience Imaging Research, Institute for Basic Science, Suwon 16419, Republic of Korea; ^g^ Department of Biomedical Engineering, Sungkyunkwan University, Suwon 16419, Republic of Korea

**Keywords:** human claustrum, 7-Tesla MRI, brain anatomy, histology, brain parcellation

## Abstract

The claustrum is a thin, bilateral structure embedded deep within the human brain. Its widespread cortical connectivity has motivated perhaps the broadest range of functional hypotheses of any subcortical structure. Yet its complex, sheet-like morphology has hindered investigation in living humans, leaving a small in vivo MRI literature marked by large and often implausible discrepancies. Here, we construct a three-dimensional histological “gold standard” model of the human claustrum and systematically evaluate three ultra-high field 7-Tesla MRI datasets against this reference and its downsampled derivatives. We show that apparent discrepancies in MRI-based claustrum morphology arise primarily from resolution-dependent effects rather than contrast limitations, which transform the claustrum’s intricate sheet into an artifactually thickened ribbon. Despite this, submillimeter MRI reliably captures a dorsal “core” containing most claustral volume and cell density and encompassing major corticoclaustral connectivity, and at the highest acquired resolution (0.5 mm isotropic), the ventral claustrum’s extension into the temporal lobe is partially recovered, with uncertainty reflecting boundary imprecision rather than anatomical absence. Together, these findings overturn the view that the human claustrum is inaccessible to MRI and establish a foundation for future functional and clinical investigation in the living human brain.

## Significance

For decades, the claustrum has captivated neuroscientists, yet progress in understanding its function in humans has been constrained by the absence of a definitive anatomical reference and the belief that the structure is too thin to image noninvasively with MRI. Here, we present a continuous three-dimensional histological model of the human claustrum, establishing a long-missing anatomical foundation for the field. By comparing simulations and 7-Tesla MRI at various resolutions against this “gold standard,” we demonstrate that submillimeter MRI captures sufficient claustral anatomy for meaningful investigation in living humans. Alongside increasing availability of ultra-high field MRI and recent advances in automated claustrum segmentation, this work establishes the claustrum as a tractable target for large-scale studies of structure, connectivity, and function.

Two decades ago, Crick and Koch argued that the human claustrum’s widespread cortical connectivity made it a candidate neural correlate of consciousness, igniting modern claustrum research (1). Since, animal work has elaborated the claustrum’s extensive connectivity (2), and inventive experiments implicate it in a diverse array of functions (3) so expansive that only basic sensory and motor processing remain outside its remit (4).

Despite this progress, the function of the human claustrum remains elusive, largely because methodological barriers have long stymied noninvasive, in vivo study. Crick and Koch argued that MRI lacked the spatial resolution required to capture the claustrum’s irregular geometry, a pessimism that has persisted despite substantial methodological advances. Indeed, the dorsal claustrum is extremely thin mediolaterally and separated from the putamen and insula by only a slender white-matter band, and though the ventral claustrum broadens as it nears both the piriform and amygdaloid complex, it exhibits lower density with cell dispersion through irregular fiber spaces (5–7). In principle, these features fall well below the nominal voxel size of structural MRI afforded by conventional and high magnetic field strengths (i.e. 1.5 and 3-Tesla), and perhaps also ultra-high field imaging (≥7-Tesla) (8).

**Fig. 1.**
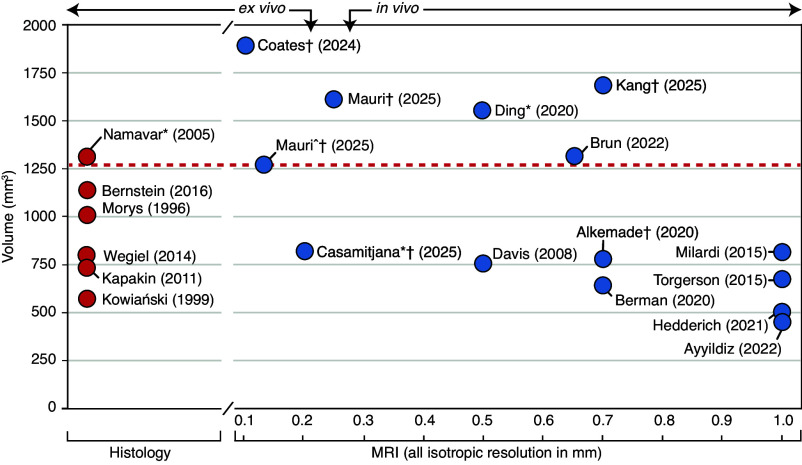
Claustrum volume estimates from past histological and MRI literature. Six histology studies (red) and 13 MRI studies (blue) provided manually or semimanually segmented claustrum volume estimates for healthy adults. Values were extracted from published tables or figures, public datasets, or by clarification by authors of published papers, and are shown as reported or available, without harmonization across methods. The gold-standard estimate provided by the present paper is shown as the red dashed line. Across MRI studies, there is more than a four-fold range between the smallest and largest reported volumes, with higher-resolution MRI yielding larger estimates (r=−0.62, P=0.016). Estimates marked with an asterisk (*) represent a single hemisphere; all others reflect the mean of both hemispheres. Estimates marked with a dagger (†) derive from 7-Tesla MRI. Estimates from Mauri (16) derive from 15 ex vivo scans spanning 0.10 to 0.25 mm isotropic resolution. Where multiple publications from the same group analyzed the same dataset, the earliest is cited.

Still, a small human MRI literature has emerged. Resolution limitations are both acknowledged and visible, with published images showing partial voluming with the surrounding capsules and nearby cortical and subcortical structures. Although few studies report quantitative measures, the 13 that provide claustrum volume estimates in healthy adults (9–26) differ by more than fourfold, exceeding expected within-subject variability (27), and diverging sharply from histology-based estimates (Fig. 1). Nonetheless, MRI has proven informative in both research and clinical contexts: diffusion and functional MRI studies have extended landmark animal findings suggesting that the claustrum is among the most highly connected structures (22, 23), and may contribute to cognitive control (28–30), pain perception (31), and higher-order processing (32), while the “claustrum sign,” a bilateral hyperintensity detectable at low resolution and field strength, has long aided diagnosis of Wilson’s disease (33) and appears to be a promising transient marker of neuroinflammation in epilepsy (34, 35).

Limitations notwithstanding, mapping the claustrum in the living human brain may ultimately require MRI: Direct human evidence is exceptionally rare, animal models face translational barriers, and both fail to definitively adjudicate between competing functional hypotheses (4). Complete bilateral absence is reported in only nine congenital cases, all with widespread atrophy and typically fatal in infancy (36–41). Acquired lesions are unilateral, incomplete, and/or nonspecific (34, 35, 42, 43). Intraoperative stimulation has produced intriguing but inconsistent effects, reflecting opportunistic electrode placement and coactivation of adjacent tissue (44, 45). Rodent models differ markedly from humans: Rodents lack an extreme capsule, complicating the insular boundary (46, 47); their endopiriform nucleus is distinct but continuous with the claustrum in humans (46); the inferior ventral “puddles” prominent in humans are poorly developed (48), and their claustrum occupies a much larger relative volume (14). Though rodents do exhibit substantial claustral-cortical connectivity (49–51), theories of human claustral function do not always extend cleanly to lissencephalic brains with fewer and less differentiated cortical areas.

The critical question is whether MRI can capture this elusive nucleus with the fidelity needed for discovery, beyond inference by analogy from animal studies and coarse disease markers. Tissue contrast is unlikely to be limiting: The claustrum is visible on T1-weighted scans despite partial voluming, consistent with its glutamatergic neurons (52) and low iron and moderate myelin content, which confer cortical-like signal properties (53). Ultra-high field scanners are increasingly common (map) and now achieve anatomical isotropic resolutions of 0.5 mm (54, 55), enabling fine-grained studies of deep brain structures (56–61). Yet even as the subcortex comes into clearer view (62), the field remains cautious, with only a handful of claustrum studies acquiring submillimeter voxels, and just two leveraging ultra-high field strength (28, 32).

One factor contributing to the lag in in vivo human MRI may be the lack of a high-resolution, three-dimensional histological reference atlas to evaluate MRI’s resolving capacity. Classical anatomical studies are richly descriptive but limited by coronal sectioning with large gaps, challenging imagination of the claustrum’s undulating course (5–7). One prior study generated a three-dimensional histological model, but it was low resolution, excluded the ventral claustrum, and exists only as photographs (13). Modern whole-brain digital atlases are more densely sampled, but delineate the claustrum de novo without specialist criteria, and diverge radically in their depiction of the ventral extent (18, 63). Two recent studies advanced the field by making publicly available claustrum segmentations from ex vivo MRI at 100 μm resolution (15, 16), but validating MRI with MRI ultimately begs our present question of if MRI can truly resolve claustral structure.

To advance the ultimate goal of elucidating human claustral function, we here address the antecedent question of whether MRI can accurately capture this elusive nucleus in vivo, using two complementary approaches. First, we segmented the BigBrain dataset (64) to create a continuous, high-resolution, histology-based three-dimensional claustrum atlas (a “gold standard”), enabling detailed morphometric description. Second, we compared this atlas and its downsampled derivatives with manual claustrum segmentations from three 7-Tesla datasets (0.5, 0.7, and 1.0 mm isotropic resolution) (55, 60, 65). Our approach disentangles spatial sampling effects from other factors, establishes resolution-specific benchmarks, and supplies the missing foundation for next-generation studies of claustral connectivity and function.

## Results

### High-Resolution Histology Reveals an Extremely Thin and Fragmented Claustrum.

**Fig. 2.**
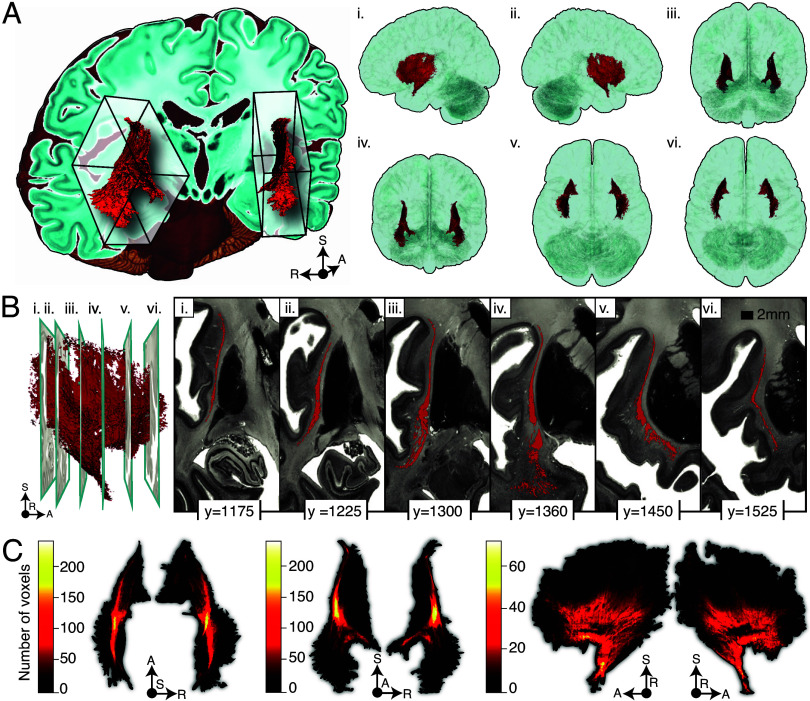
Histological gold-standard reconstruction of the human claustrum. (*A*) *Left*: Bilateral claustra of the gold-standard model (red) shown within the BigBrain dataset, with oriented bounding boxes illustrating oblique orientation relative to cardinal axes. *Right*: Six canonical views (left, right, posterior, anterior, inferior, superior) highlight the claustrum’s shape and position within the brain. (*B*) *Left*: Lateral view of the right claustrum with six posterior-to-anterior coronal slice positions indicated (*i*–*vi*). *Right*: Corresponding coronal slices with segmentation overlays (BigBrain coordinates provided), illustrating marked variability, including alignment with the insular cortex posteriorly (*i* and *ii*), fragmentation into ventral “puddles” at mid-depth (*iii* and *iv*), and curvature around the putamen anteriorly (*v* and *vi*). (*C*) Two-dimensional projection maps of the gold-standard claustrum in axial, coronal, and sagittal planes, for the left and right claustra, respectively, with color indicating voxel count as a proxy for thickness (dark = low count, light = high count). A truncated scale is used for the sagittal view to enable visual distinction. The thickest regions, represented by the bright yellow and red “core,” are comparable to the claustrum’s appearance in submillimeter MRI (Fig. 4*B*) and are reliably identified across participants (*SI Appendix*, Fig. S4).

Drawing on the exceptional anatomical detail of the 100 μm BigBrain dataset, we manually delineated a continuous bilateral segmentation of the human claustrum surpassing the detail of existing histological atlases (Fig. 2 *A* and *B*). The resulting “gold standard” model recapitulates defining features described in classical literature: dorsally, an exquisitely thin sheet follows the insular convolution and bends laterally over the central insular sulcus; ventrally, the claustrum broadens into a reticular arrangement, fragmenting into small “puddles” separated by white-matter laminae in the anterior temporal lobe.

To ensure that our segmentation closely approximated features visible at higher histological resolution, we compared challenging regions and regular intervals against the BigBrain at 20 μm and 1 μm in-plane resolution. The two greatest challenges at 100 μm reflected features that proved difficult to resolve even at cellular resolution: We could not detect tiny islands abutting the piriform cortex near the terminal zone of the lateral olfactory tract (5, 11), and some boundaries with the amygdaloid complex in the anterior ventral claustrum were ambiguous (7, 66). To appreciate individual variability, we qualitatively compared our BigBrain segmentation to claustral mapping of additional donor brains in the Julich Brain Atlas dataset (67). Consistent with prior anatomical descriptions, the ventral claustrum was notably variable in puddle characteristics and relation to adjacent structures; nonetheless, BigBrain’s claustral anatomy appeared broadly representative of the range observed across brains. Also, as no histological resolution of BigBrain nor the ten Julich brains revealed a clear structural basis for delineating putative claustral subsections—and it is unclear whether such subdivisions can be reliably distinguished on cytoarchitectural grounds alone (68)—we adopted the rhinal sulcus as a practical heuristic to separate dorsal from ventral claustrum (69).

BigBrain’s continuous reconstruction and our full segmentation enable more precise morphometry than interpolation across sectioned histology. Bilateral centers of mass were symmetric at approximately ±32 mm from midline, 1 mm posterior and 5 to 6 mm inferior to the anterior commissure line. The principal axes showed an oblique trajectory, with anterior (40^°^) and inferior (50^°^) deviation relative to canonical neuroanatomical planes. Three-dimensional measurements averaged across hemispheres are presented in *SI Appendix*, Table S1 (hemisphere-specific results in *SI Appendix*, Tables S2 and S3). Total claustrum volume was 2,536.02 mm^3^ (left: 1,325.58 mm^3^; right: 1,210.44 mm^3^), approximately 0.13% of the total brain volume, including cerebellum and ventricular CSF. Averaged maximal axis-aligned extents measured 28.35 mm mediolaterally, 53.45 mm anteroposteriorly, and 55.45 mm superoinferiorly. Shape descriptors indicated low roundness and high flatness, consistent with an elongated planar structure.

**Fig. 3.**
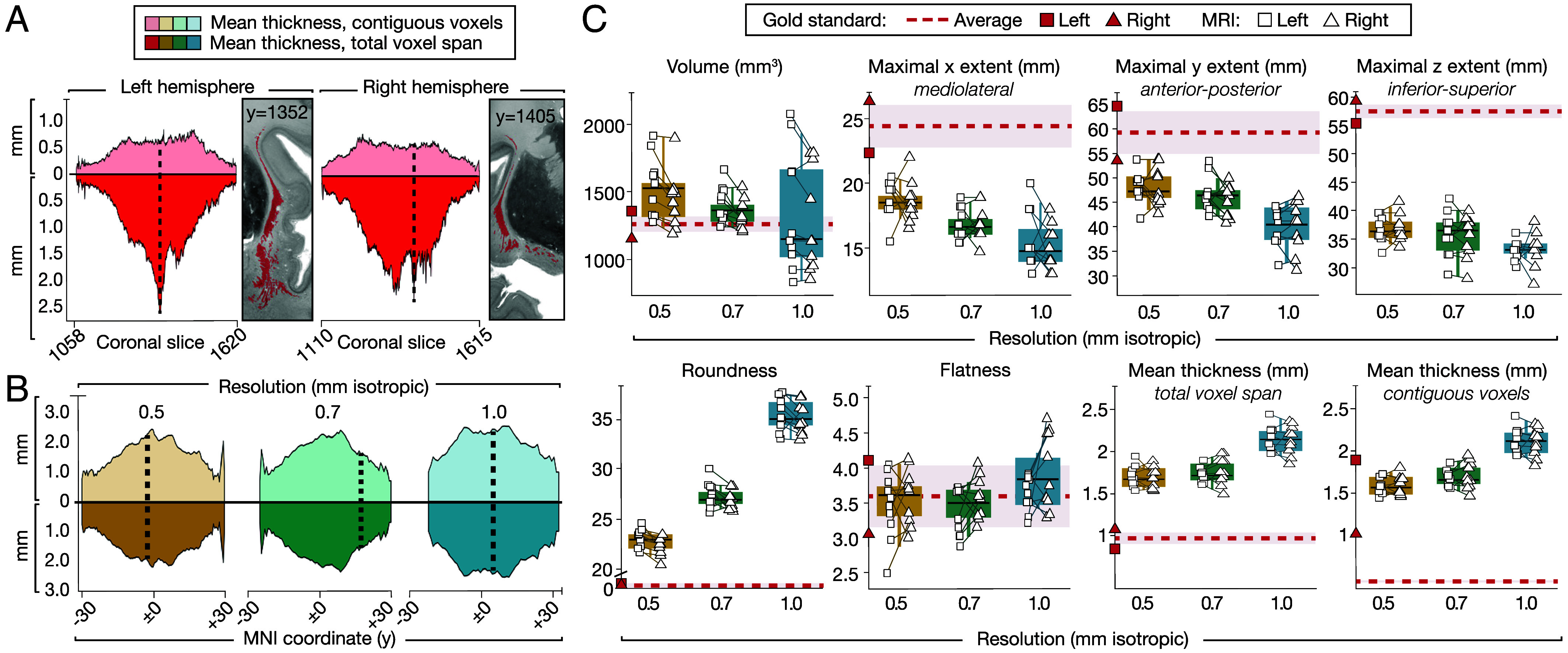
Resolution-dependent changes in claustrum morphology. (*A*) Slice-wise thickness profiles of the histological gold standard. In each silhouette plot, mean thickness based on contiguous voxels (*Upper*, pastel) and total voxel span (*Lower*, solid) are mirrored about the horizontal axis. The dashed black line marks the coronal slice showing the maximal discrepancy between measures; corresponding histological sections are shown in the *Insets*. Discrepancies reflect ventral “puddles” fragmented by intervening white matter. (*B*) Mean slice-wise thickness profiles for the three MRI datasets, averaged across participants and hemispheres. Profiles are more symmetric than for the gold standard, reflecting the absence of ventral fragmentation at MRI resolutions, with thickness ratios approaching unity. (*C*) Estimates of eight morphometric measures for MRI datasets (boxplots) relative to the histological gold standard (red dashed line). Squares and triangles denote left and right hemisphere values, respectively; boxplots show hemisphere-averaged values with medians indicated by solid horizontal lines. All measures exhibit clear resolution dependence except volume, illustrating the volume paradox.

To characterize the claustrum’s thinness and ventral fragmentation, we computed two-dimensional (slice-wise) thickness metrics to mitigate overestimation of maximal three-dimensional extents. Across coronal slices, the mean span of mediolateral thickness was 1.21 mm ± 1.39 mm, whereas the thickness of contiguous voxels was just 0.56 mm ± 0.52 mm (Fig. 3*A*). Discrepancies between these measures occurred in >90% of slices and exceeded a twofold difference in 40%, indicating interruption of white matter fibers, primarily in regions containing ventral “puddles.” All coronal slices contained submillimeter spans, and 85% contained at least one location only one voxel thick (100 μm). Thickness maps projected along orthogonal planes revealed that though the dorsal claustrum forms a narrow sheet, it contains a relatively cohesive central “core,” whereas the ventral claustrum, despite its broad mediolateral span, contains fewer claustrum voxels due to punctuation by white matter (Fig. 2*C*).

### Downsampling Systematically Alters Claustral Morphometry.

To assess how spatial resolution affects claustrum morphometry, we downsampled the histological gold standard to isotropic resolutions ranging from 0.4 to 2.0 mm in 0.1 mm increments, spanning the full continuum of whole-brain structural MRI from the current upper bound achievable at ultra-high field to resolutions typical of 3-Tesla acquisition, and across binarization thresholds (0.2 to 0.8), reflecting liberal to conservative segmentation styles. Resolution exerted heterogeneous effects, but the resulting degradation was predictable, with resolution alone explaining more than 93% of variance across eight morphological metrics (*SI Appendix*, Fig. S1). Volume was largely insensitive to voxel size (P=0.18). However, all three maximal axis-aligned extents decreased systematically with coarser resolution (i.e., larger voxel size; all pFDR < 0.01), shrinking by 0.16 mm mediolaterally, 0.19 mm anteroposteriorly, and 0.40 mm superoinferiorly per 0.1 mm increase in resolution. Roundness increased at lower resolution, while flatness remained stable except at the lowest resolutions and highest thresholds, where it dropped sharply, reflecting a shift toward a less elongated and planar claustral geometry (both pFDR < 0.01). Consistent with partial voluming, both total voxel span and contiguous voxel thickness increased at coarser resolution (pFDR < 0.01).

### MRI Partially Captures Claustral Anatomy.

**Fig. 4.**
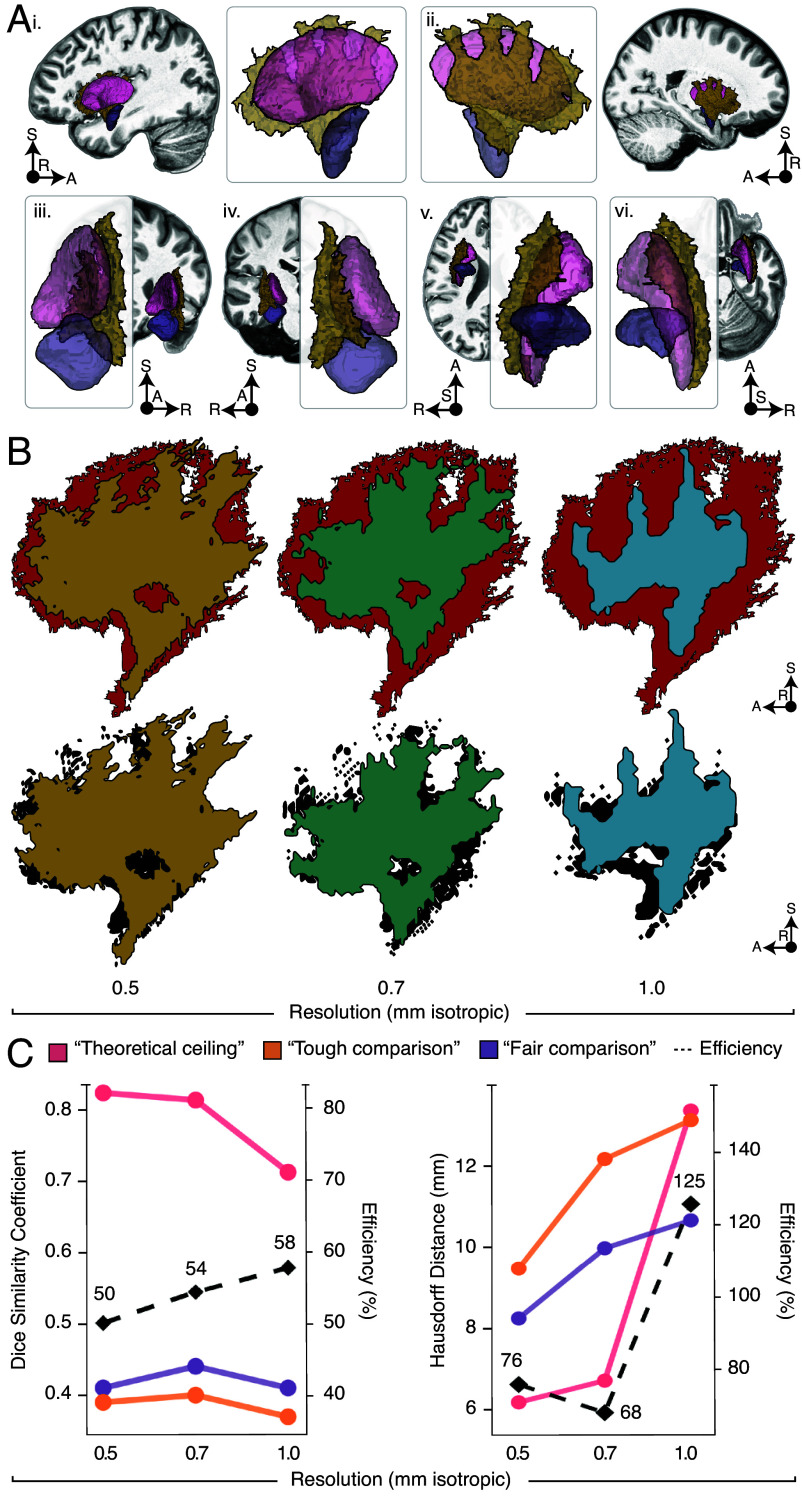
MRI performance relative to histological and resolution-matched gold standards. (*A*) Manually segmented claustrum (yellow) from a representative participant in the 0.5 mm MRI dataset shown in six canonical views (*i*–*vi*: Left, Right, posterior, anterior, inferior, and superior), alongside the putamen (pink) and amygdala (purple) as labeled by the Xiao atlas. Background slices correspond to the nearest slice not containing claustral voxels. Among the MRI datasets, only the 0.5 mm resolution reliably and unambiguously distinguishes all three structures. (*B*) Spatial overlap of MRI segmentations (color) with either the histological gold standard (*Top*; red; “tough comparison”) or the resolution-matched downsampled gold standard binarized at a 50% threshold (*Bottom*; black; “fair comparison”). Lateral views of the left hemisphere are shown for a representative participant with median claustrum volume. Across resolutions, the central core of the claustrum is consistently recovered, whereas peripheral boundaries are progressively lost, indicating impaired boundary precision rather than gross mislocalization. (*C*) Spatial agreement quantified using Dice similarity coefficient (*Left*) and Hausdorff distance (*Right*). Orange lines show MRI performance relative to the histological gold standard (“tough comparison”), and purple lines show performance relative to the resolution-matched downsampled gold standard (“fair comparison”). Pink lines indicate the theoretical ceiling (downsampled versus full-resolution gold standard), and dashed black lines indicate MRI efficiency, defined as the proportion of achievable performance attained at each resolution. At submillimeter resolutions, MRI captures approximately half of the maximal possible volumetric overlap (DSC) and 68 to 76% of attainable boundary precision (HD). At 1.0 mm, reduced ceiling performance inflates HD efficiency despite poorer absolute boundary accuracy.

In all three ultra-high field T1-weighted MRI datasets (0.5, 0.7, and 1.0 mm isotropic resolutions), the claustrum appeared hypointense with adequate contrast-to-noise ratio (CNR) relative to surrounding white matter (0.5 mm = 4.42 ± 0.52, 0.7 mm = 3.29 ± 0.39, 1.0 mm = 2.78 ± 0.60). Though raters reported lower confidence in boundary delineation compared to histology, interrater agreement remained high (all DSC > 0.9, see *SI Appendix*, Fig. S2). The claustrum’s proportion of intracranial volume was consistent across datasets: 0.27 ± 0.04 at 0.5 mm, 0.26 ± 0.04 at 0.7 mm, and 0.25 ± 0.09 at 1.0 mm, and centers of mass were likewise stable (maximum difference 1.67 mm; *SI Appendix*, Table S4). However, only the 0.5 mm dataset enabled unambiguous differentiation in all participants, with manually drawn claustrum segmentations sometimes abutting but never overlapping adjacent cortical or subcortical structures, despite partial voluming with white matter (Fig. 4*A*).

Comparison of claustral morphometry across MRI datasets is visualized in Fig. 3*C* and quantified in *SI Appendix*, Table S5. As in the downsampling simulation, claustrum volume was not significantly affected by resolution (pFDR = 0.25), but all other metrics showed significant resolution effects, from which two patterns emerged. First, the 1.0 mm dataset showed systematically greater divergence from the submillimetric datasets: Post hoc tests showed that the 0.5 mm and 0.7 mm datasets differed only occasionally (3 of 7 significant comparisons), whereas the 1.0 mm dataset differed from both submillimetric datasets across all significant metrics. Second, measurements were generally less stable at 1.0 mm resolution, with volume exhibiting markedly high instability (CV = 0.31; see *SI Appendix*, Table S6).

As anticipated, direct comparison between MRI segmentations and the histological gold standard revealed substantial deviations across most morphometric measures (Fig. 3*C* and *SI Appendix*, Table S7). Both submillimeter MRI datasets overestimated claustrum volume, most prominently at 0.5 mm; the 1.0 mm dataset did not differ significantly. Flatness was the sole metric that remained stable across all resolutions, reflecting proportional shrinkage along the anteroposterior and superoinferior axes. All other measures showed significant resolution-dependent deviations that increased with coarser resolution. Two-dimensional thickness estimates showed especially large discrepancies (total span +74 to 121%; contiguous thickness +234 to 344%). Fidelity was poorest in the middle third of the anteroposterior axis where the ventral claustrum broadens and fragments into “puddles:” The gold standard’s span-to-contiguous-thickness ratio (2.76) collapsed to near-unity at all MRI resolutions, indicating near-complete loss of anatomical detail (Fig. 3 *A* and *B* and *SI Appendix*, Table S8).

Spatial agreement between MRI and the gold standard indicated poor correspondence (*SI Appendix*, Table S9). Dice coefficients were uniformly low (DSC 0.37 to 0.40), while Hausdorff Distances were high and increased with coarser resolution (HD 9.49 mm to 13.05 mm). When boundary uncertainty was accommodated using adjusted metrics (dilated DSC and balanced average HD), spatial agreement improved substantially, indicating that MRI-histology discrepancies reflected boundary imprecision rather than gross mislocalization (Fig. 4*B*).

### MRI Capture Approaches Theoretical Resolution Limits.

Finally, each MRI dataset was evaluated against its resolution-matched downsampled gold standard binarized at a 50% threshold, which we took as the theoretical maximum detail recoverable at a given resolution (*SI Appendix*, Table S10). Again, MRI consistently overestimated volume, albeit most strongly at 1.0 mm (+53.34%). Maximal mediolateral and superoinferior extents were truncated across all MRI datasets, while anteroposterior extent was significantly longer at 0.5 mm (+6.29%) but comparable at 0.7 mm and 1.0 mm. Roundness showed mild inflation, and flatness remained stable except for an increase at 1.0 mm (+22.42%). Slice-wise thickness was consistently overestimated, with the largest deviations observed at coarser resolutions (total span +40 to 48%; contiguous thickness +53 to 67%).

In addition to spatial agreement metrics, we computed ‘efficiency’ as the proportion of agreement attainable given the downsampled gold standard’s inherent ceiling (Fig. 4*C* and *SI Appendix*, Table S11). DSC was low and resolution-invariant (0.41 to 0.44), corresponding to 50.00% to 57.75% of theoretically achievable volumetric overlap. HD ranged from 8.2 mm to 10.65 mm, representing 68.14% to 75.75% of attainable boundary precision at submillimeter resolutions; at 1.0 mm, however, the downsampled gold standard exhibited such poor boundary definition that MRI performance nominally exceeded the ceiling (124.69%), underscoring that claustral boundaries are poorly represented at “conventional” resolution.

### Interindividual Variability, Hemispheric Asymmetry, and Sex Differences.

Despite morphometric distortion, MRI is arguably the best tool for studying claustral variation in vivo. Thus, we pooled the three MRI datasets to explore individual variability, hemispheric asymmetry, and sex differences, while acknowledging inherent measurement limitations.

#### Individual variability.

A probabilistic overlay constructed from all 30 MRI segmentations revealed high spatial agreement in the dorsal “core” of the claustrum and progressively lower agreement toward the ventral extent (*SI Appendix*, Fig. S3).

#### Hemisphere differences.

In the pooled MRI sample, the right claustrum was significantly larger in volume (pFDR < 0.01, d = 0.91) and exhibited greater flatness (pFDR = 0.03, d = 0.47), whereas the left claustrum was more round (pFDR < 0.01, d = 0.75). In the 0.5 mm dataset, participant-level asymmetry indices (AI) confirmed significant hemispheric asymmetry in volume (AI =−0.036, pFDR = 0.019) and roundness (AI = 0.021, pFDR = 0.014). Asymmetry patterns were consistent across resolutions.

#### Sex differences.

Comparison between sexes revealed no significant differences on any morphometric measure, between or across hemispheres; likewise, controlling for intracranial volume (ICV) revealed no significant effects of sex or ICV.

## Discussion

Despite decades of interest in claustral function, investigation in living humans has remained elusive because of pessimism that MRI’s spatial resolution is inadequate to capture the structure’s unusual geometry. Here, we ask whether MRI can resolve the claustrum with sufficient fidelity to support in vivo study, by characterizing its anatomy using complementary approaches: high-resolution histology (a 100 μm BigBrain-derived gold standard model) and ultra-high field MRI (7-Tesla datasets at 0.5 mm, 0.7 mm, and 1.0 mm isotropic resolution). Through systematic comparison of these modalities and resolution-matched simulations, we establish what each captures of claustral structure, quantify the limits of in vivo imaging, and provide a histology-based benchmark that lays the foundation for next-generation studies of human connectivity and function.

### A Three-Dimensional Histological Gold Standard for the Human Claustrum.

The BigBrain-derived histological gold standard model provides a continuous three-dimensional reconstruction of the human claustrum derived directly from serial histological sections, without statistical interpolation. This publicly available, interactive model enables appreciation of claustrum size, complexity, and anatomical relationships in a way that traditional illustrations and photographs cannot (Fig. 1). The model also highlights striking architectural contrasts: Although the claustrum is often only a few hundred microns thick, it spans more than 5cm anteroposteriorly and superoinferiorly, with a total bilateral volume twice that of the substantia nigra and approaching three-quarters that of the amygdala (70). Its large extent belies common descriptions of the claustrum as a “tiny” nucleus (71), but its thinness and undulation helps explain why many have assumed it to be beyond the reach of conventional MRI.

The gold standard model resoundingly accords with qualitative descriptions and illustrations of classical anatomical literature (5–7), though direct comparison is limited by sparse reporting of quantitative metrics and pronounced methodological differences, including fixed versus fresh tissue and varying conversion factors. Our bilateral volume lies at the upper end of published histological estimates (Fig. 1): Only one reports a slightly higher value (9), whereas five report smaller volumes (10–14). Only one prior study quantified extents and reported a substantially shorter anteroposterior and dorsoventral span but larger mediolateral span, likely reflecting coarser sampling (13). We attribute the comparatively larger measurements of the gold standard to our complete segmentation of the entire claustrum, made possible by BigBrain’s high tissue integrity and visual contrast.

### Anticipated Resolution-Driven Distortion.

Downsampling the gold standard model isolates resolution-driven distortion and establishes a critical interpretive guardrail for MRI: Assuming cell-stained histology affords equal or better identification of claustral tissue than voxelized MRI, any anatomical feature that disappears in downsampling simulations may not be reliably detected in MRI at the corresponding resolution, regardless of apparent visualization. As expected, discrepancies were greatest at the lowest resolutions and most conservative thresholds (*SI Appendix*, Fig. S1), but even the highest MRI-like resolution and most liberal thresholds fundamentally altered claustral morphology and struggled to preserve the ventral claustrum. Simultaneously, the superoinferior extent was disproportionately truncated—reflecting loss of the ventral portion extending into the temporal lobe—yet in regions that remained resolved, contiguous slice-wise thickness inflated as small gaps were bridged and isolated “puddles” merged. Roundness increased as thin edges disappeared, while flatness remained stable until the coarsest resolutions artifactually inflated mediolateral thickness and eliminated sheet-like geometry. This progressive degradation explains why even high-resolution MRI studies typically visualize the claustrum as a simplified ribbon lacking ventral extension.

A notable consequence of this degradation was a “volume paradox” (*SI Appendix*, Fig. S1): Despite marked truncation of the claustrum’s anteroposterior and dorsoventral extents, total volume remained statistically stable across resolutions because the reduction in extents was offset by partial-volume inflation of mediolateral thickness. Thus, volume stability does not indicate preserved anatomy but rather that volume is an insufficient descriptor of claustral morphology. This phenomenon is also described in other thin structures where boundary voxels disproportionately influence total volume (72, 73). Further, downsampling to 1.0 mm with a 50% threshold (optimistically representing the most common in vivo MRI resolution and a typical “majority-vote” segmentation approach), produced substantial divergence from histological ‘reality’ across nearly all morphometric measures. This implies that MRI at conventional resolution characterizes substantial partial volume-induced measurement error alongside anatomy, raising questions about interpretation of much of the extant 3-Tesla literature at or near this resolution (Fig. 1).

### In Vivo Claustrum Capture With Ultra-High Field, High-Resolution MRI.

We next quantified the extent to which the claustrum, as illuminated by the gold standard model, can be captured in vivo. Three ultra-high field datasets established that the claustrum can be (at least partially) identified using standard whole-brain MP2RAGE protocols feasible at most 7-Tesla centers, requiring no specialized contrast (74). Partial volume effects, evident as intermediate signal intensities at tissue interfaces and loss of anatomical detail, were apparent at all resolutions but more pronounced as voxel size increased; this is expected given that increasing isotropic spatial resolution from 1.0 mm to 0.7 mm and 0.5 mm decreases volume by factors of approximately 3 and 8 (1,000 nL to 343 nL and 125 nL) (75). The 0.5 mm dataset uniquely separated the claustrum from surrounding structures (Fig. 3*A*), though at all resolutions, at least one participant exhibited some degree of apparent ventral “dropout,” almost certainly artifactual rather than true absence given histology’s consistent demonstration of ventral claustrum (67), albeit with some shape and density variability (6). No aspect of the claustrum exhibited markedly different contrast properties despite known variation in neuronal density (14).

As in downsampling, MRI showed paradoxical volume stability across datasets despite shape changes (Fig. 2*B*), underscoring that classical sampling adequacy criteria such as the 5% voxel-to-ROI volume guideline, satisfied at all resolutions, prove misleading for thin structures (72). This stability contrasts sharply with dramatic between-study variability in the literature, with higher-resolution studies tending to report larger volumes (Fig. 1). The most likely explanation is discrepancy in segmentation style: The claustrum’s extreme thinness makes measurements highly sensitive to boundary decisions. Illustratively, though we implemented the manual segmentation protocol of Kang et al. (19) on resolution-matched data, we obtained bilateral volumes 20% smaller than theirs. Likewise, independent groups (15, 16) segmenting the same ex vivo brain (76) reported volumes differing by 18% in one hemisphere. Rigorous standardization of manual segmentation or automated algorithms are needed; we recommend reporting standards to make claustrum findings interpretable and comparable across studies (*SI Appendix*,*Discussion* and section 1).

While decades of MRI-based claustrum research have acknowledged potential limitations, none have tested these assumptions against a histological reference, creating an evidence base of uncertain reliability. Comparison of MRI to the gold standard revealed poor spatial agreement (Fig. 3*B*) and substantial deviations across all morphometric measures, except paradoxically stable volume (Fig. 2*C*). Nevertheless, all resolutions showed a “parochial” detection pattern: MRI reliably captured thick core regions while losing thin peripheral features. The preserved core corresponded to thick mid-dorsal regions in the histological map (Fig. 1*C*), whereas thin boundaries escaped detection, including much of the ventral claustrum but also superior dorsal aspects where the claustrum bends over the putamen. Given the disproportionately incomplete capture of the ventral claustrum at current MRI spatial resolution, studies capturing this region should interpret findings cautiously and account for the likely contribution of partial voluming, pending advances in resolution affording more reliable ventral capture. When boundary uncertainty was accommodated using adjusted metrics appropriate for thin structures (dDSC and baHD), overlap was reasonable at both submillimeter resolutions.

Importantly, the claustrum’s thickest dorsal portions account for most cell density and volume (14), and house primary connectivity to sensorimotor and frontal association cortices (2, 77, 78), grounding distinct and prominent hypotheses of claustral function (3, 79, 80). What functional insight may be lost given incomplete ventral capture remains an open question. In humans, the ventral claustrum projects most strongly to limbic, olfactory, and temporal regions (3), suggesting possible roles in emotional processing and memory. Though comparative insights may prove challenging, ventral “puddles” are apparent across several large-brained mammals (14), though their precise shape and location vary markedly across species, potentially reflecting optimization of projection distances and wiring length (81). Other human subcortical research has succeeded under such constraints: Hippocampal studies focus on CA1 and dentate gyrus while accepting poor CA2/CA3 resolution (82), and substantia nigra work routinely targets ventral tiers despite dorsal detection failures (83). Such constraints have not stymied progress but have prompted greater anatomical precision, more targeted hypotheses, and appropriately cautious interpretation: A mature scientific approach the claustrum field now requires.

### Disentangling Spatial Sampling from Other MR Limitations.

Next, we asked if MRI’s limitations reflect resolution constraints or other technical factors, so compared each MRI dataset to its resolution-matched downsampled gold standard binarized at 50% threshold (Fig. 3*B*). This “fair comparison” isolates spatial sampling effects from other potential sources of discrepancy such as the sensitivity of MRI contrast to histologically determined cell density. We found that MRI distorts claustral anatomy through mechanisms largely, but not entirely, explained by spatial sampling. Submillimeter MRI achieved approximately half of theoretically achievable volumetric overlap and the majority of attainable boundary precision (Fig. 3*C*). Importantly, our MRI datasets were not optimized for claustral nor subcortical capture, suggesting the 25 to 50% efficiency shortfall may reflect correctable technical factors rather than fundamental limits. Potential optimization could include tuning inversion time for claustral contrast, testing slice-plane angulation relative to the insular sheet, exploring modest anisotropy as used for other small subcortical structures (84), and testing alternative contrasts that may enhance capsule boundaries and have been useful to automated segmentation efforts (16, 85).

Of course, some differences between histology and MRI reflect inherent cross-modal differences between cell staining and image contrast, and are better appreciated by comparison of histology to high-resolution ex vivo MRI. Two independent annotations (15, 16) of the same 100 μm ex vivo 7-Tesla MRI dataset (76)—one using multiplanar, multirater segmentation with union smoothing, the other a sparse single-rater coronal approach with interpolation—yielded deviations from the gold standard, including inflated volumes, truncated extents, higher roundness, and span-to-contiguous ratios near unity (*SI Appendix*, Table S12). These discrepancies mirror those observed in our in vivo MRI and downsampling analyses, suggesting they may arise from MRI contrast properties rather than spatial sampling alone. Thus, though technical optimization may improve claustral imaging within existing 7-Tesla infrastructure, cell-stained histology remains the necessary reference for precise anatomical characterization.

### Implications for Functional MRI Studies of the Claustrum.

How do these structural results bear on studies of claustral function? Even at ultra-high field strength, typical fMRI resolution (1 to 2 mm) falls far below the submillimeter resolution required for reliable claustral localization—voxels containing predominantly claustral tissue. Echo-planar imaging further degrades effective resolution through T2* blurring, susceptibility-induced distortion, and corrective resampling artifacts, with additional confounds likely arising from insular perforators of the middle cerebral artery and venous drainage (86).

Despite these challenges, two pioneering 7T fMRI studies provide proof-of-concept that claustral signal can be functionally isolated, and their contrasting approaches reflect a fundamental trade-off between sensitivity and specificity. Krimmel et al. (28) took a conservative approach: The claustrum was manually drawn on individual structural scans and flanking insula and putamen signal was regressed out, recovering resting-state correlations at 1.5 mm isotropic resolution. Restricted to what is individually visible on structural MRI, this approach maximizes anatomical specificity at the cost of sensitivity to thinner and ventral claustral regions. Coates et al. (32) took a more liberal approach exploiting the principle that voxels containing claustral tissue can generate measurable BOLD signal despite partial voluming (87): A claustrum label (15) derived from a 100 μm ex vivo MRI dataset (76) was warped into individual subject space, enabling detection of task-evoked visual responses at 1.34 × 1.34 × 0.8 mm resolution in the ventral claustrum. This maximizes sensitivity to the full claustral extent, including regions invisible on structural scans, at the cost of reduced certainty that BOLD signal originates exclusively from claustral tissue.

To support future functional investigation, we provide a cross-modality probabilistic atlas integrating the histological gold standard, the 0.5 mm 7T dataset (n=10), and two high-resolution MRI segmentations from Mauri et al. (16), one ex vivo at 100 μm (76) and one in vivo at 250 μm (88) (*SI Appendix*, *Methods* and section 1). Anchored in cell-stained histology and integrating cross-modal variability across 13 brains, it provides a versatile anatomical prior that explicitly encodes boundary uncertainty across the full claustral extent. Its high-agreement dorsal core supports conservative, hypothesis-driven analyses; the full atlas extent supports exploratory investigation, guiding seed placement in resting-state fMRI and interpretation of apparent white matter activations in task fMRI.

### Exploratory Analyses Suggest Modest Hemispheric Asymmetry.

Finally, exploratory analyses across the three MRI datasets revealed reproducible patterns despite demonstrated limitations of in vivo resolution. The probabilistic overlay showed highest spatial agreement in the dorsal midsection, with increasing variability toward the superior, anterior, and ventral periphery where partial voluming was most pronounced (*SI Appendix*, Fig. S3). The extent to which apparent interindividual differences reflect genuine anatomical variability versus partial voluming remains uncertain; the latter is nonetheless the more parsimonious explanation, as our downsampling simulations demonstrate that the fine-scale features most susceptible to interindividual variability—namely ventral puddle characteristics and peripheral boundaries, as observed across the Julich Brain Atlas dataset (67)—are precisely those lost at current MRI resolutions. Hemispheric asymmetry was modest but consistent: The right claustrum appeared larger and flatter, the left smaller and rounder. This contrasts with the leftward bias in the histological gold standard. Prior MRI reports are mixed: Some report rightward trends in adults (19, 22, 24, 26), significant rightward effects in adolescent males (89) and neonates (90), and others report nonsignificant (20) or significant leftward effects (23, 91). Two independent segmentations of the same 100 μm ex vivo MRI (76) also found a larger right claustrum (15, 16). No significant sex differences emerged, consistent with most prior studies showing absent effects (24) or higher male volumes that disappear after ICV adjustment (19) or do not reach significance (22), though one study found higher female volumes after ICV adjustment (26), and subtle tissue-composition differences have been reported (20, 92). Collectively, these results support pooling sexes and modeling hemispheres separately to maximize statistical power.

### Limitations.

Our approach has several limitations. The BigBrain-derived gold standard is from a single 65-y-old male brain, limiting assessment of population variability. The 100 μm BigBrain smooths some claustral features visible in the 20 μm and 1 μm versions (67), but was used due to feasibility, its availability in MNI space, and its widespread adoption. Manual segmentation, including the use of different raters by hemisphere, introduces some subjectivity despite high interrater reliability. Nonetheless, pending higher-resolution, multidonor, and multimodal validation, this remains the most complete three-dimensional histological model of the human claustrum available.

Our MRI analysis used three convenience datasets (each n=10) acquired on different Siemens systems with slightly varying protocols, introducing potential site and sequence heterogeneity, though we observed no substantial SNR limitations or distortion artifacts. Participant-related biases cannot be excluded, but demographics were comparable across datasets, and prior subcortical atlasing suggests that morphological estimates stabilize with modest samples (>5) (93–95). Participants were younger than the BigBrain donor, although current evidence suggests some age effects on claustral morphometry in late adulthood (26, 53). This study did not analyze 3-Tesla data, but because gray-white matter contrast in T1-weighted imaging does not change substantially with field strength, the limitations we identify are driven primarily by spatial resolution rather than field strength per se; thus, the resolution-specific benchmarks established here are therefore directly informative for lower-field MRI. The strengths of this analysis lie in its use of whole-brain sequences that most 7-Tesla centers can implement, and are increasingly available in public datasets.

### Conclusions and Outlook.

For more than two decades, the claustrum has been among the most intriguing structures in neuroscience and the subject of influential speculation about its function, yet it has remained relatively underinvestigated using MRI, lacking rigorous morphological characterization and leaving human work comparatively limited alongside a predominantly animal-based literature. The present study provides a continuous three-dimensional histology-based model of the claustrum and a systematic test of MRI’s ability to capture it. Our results challenge the view, persisting since Crick and Koch, that the claustrum is a tiny nucleus beyond resolve. Submillimeter 7-Tesla MRI recovers more than half of theoretically attainable anatomical detail, reliably capturing the thick dorsal core that comprises most claustral volume and houses major corticoclaustral connectivity hubs (2). Ventral “puddles” remain challenging, yet at 0.5 mm isotropic resolution their overall extent is partially preserved, with uncertainty arising from boundary imprecision rather than complete anatomical loss. The current state-of-the-art of in vivo MRI permits productive investigation of claustral structure and cautious exploration of its function (15, 28), positioning the field for a new phase of investigation that may answer long-standing questions about the claustrum’s contribution to human cognition.

## Materials and Methods

### Histological Dataset.

The BigBrain dataset is a super-high-resolution digital reconstruction of histological sections from a 65-y-old male with no known neurological or psychiatric conditions at the time of death (64). Tissue was stained using a modified silver impregnation method based on Merker’s technique, providing high contrast for neuronal cell bodies and cytoarchitectural analysis. We selected BigBrain after reviewing publicly available high-resolution digital ex vivo datasets, including the MGH atlas (76) and the Allen Brain Atlas (18), as the claustrum was the most visually distinct. To enable direct comparison with in vivo MRI, we used the 100 μm isotropic resolution voxelized version provided in “BigBrain3D Volume Data Release 2015” (https://ftp.bigbrainproject.org/bigbrain-ftp/), aggregating the original 20 μm reconstruction of 7,404 histological sections, which includes corrections for tissue shrinkage and is aligned to MNI-ICBM152 2009b symmetric space.

### Histological Segmentation Approach.

Full details of claustrum localization and segmentation are provided in *SI Appendix*, *Methods* and section 2. To enable real-time navigation of the high-resolution histological volume, hemisphere-specific subvolumes encompassing the claustrum and surrounding structures were extracted. Manual segmentation of the claustrum as a single structure was performed in ITK-SNAP (96), using a one-voxel-sized brush and Wacom tablet. Claustral tissue was identified as low-intensity (dark) voxels relative to surrounding white matter, assessed simultaneously in all three orthogonal planes and as a three-dimensional volume. Optimized viewing planes were emphasized to account for regional visibility, with the dorsal claustrum segmented primarily in the axial plane, the ventral claustrum in the coronal plane, and the sagittal plane used primarily to validate the posterior temporal claustrum (19). Voxels were included as claustral irrespective of spatial continuity or cluster size, provided they met anatomical criteria and did not overlap adjacent structures (*SI Appendix*, Fig. S4), with ambiguous regions cross-referenced against the BigBrain dataset at 20 μm and 1 μm in-plane resolution (67). Prior to full manual segmentation, several interpolation-based methods were tested (97–99), but were not pursued due to inadequate performance, particularly in ventral regions inferior to the fundus of the rhinal fissure (*SI Appendix*, Fig. S6). The right hemisphere was segmented by one rater (SP) and the left by another (NC), following an eight-step quality control process detailed in *SI Appendix*, *Methods* and section 3, yielding high interrater agreement (DSC 0.87 to 0.93, see *SI Appendix*, Fig. S5).

### MRI Datasets.

Three public in vivo 7-Tesla MRI datasets with isotropic resolutions of 0.5 mm, 0.7 mm, and 1.0 mm were analyzed, each comprising 10 healthy adult participants. The 0.5 mm “MICA-PNI” dataset was acquired in Montreal, Canada (55), and the 0.7 mm (60) and 1.0 mm (65) datasets were acquired at Maastricht University, The Netherlands. All participant data were fully deidentified prior to use; ethical approval and informed consent procedures for each dataset are described in the original publications. All participants were healthy adults with no history of major neurological illness. Demographic details are provided in *SI Appendix*, Table S13. These resolutions were chosen to span the practical range of whole-brain in vivo structural MRI, with 0.5 mm representing the upper bound presently achievable within reasonable scan times (≤15 min); 0.7 mm representing recent, large public datasets (21); and 1.0 mm remaining the most typical resolution reported in the literature, including recent claustrum studies (Fig. 1).

### MRI Acquisition.

The 0.5 mm dataset was acquired on a Siemens 7-Tesla Magnetom Terra (Siemens Healthineers, Erlangen, Germany), using a 32Rx/8Tx head coil (NOVA Medical Inc., Wilmington, MA, United States). Three runs were obtained at separate time points over an average span of 96.45 (±74.71) days. The 0.7 mm and 1.0 mm datasets were acquired in one run on a Siemens 7-Tesla Magnetom using a 32Rx/1Tx head coil (NOVA Medical Inc., Wilmington, MA, United States). From all datasets, we utilized whole-brain 3D-MP2RAGE uniform (UNI) images (T1-weighted) (100), on which the claustrum appears hypointense. Acquisition details are provided in Table 1; preprocessing and processing steps are detailed in *SI Appendix*, *Methods* and section 4.

**Table 1.** MRI acquisition parameters

Dataset resolution (mm isotropic)	TE (ms)	TR (ms)	Flip angle (^°^)	TI_1_/TI_2_ (ms)	Scan length (m:s)	Acceleration factor (PE)
0.5	2.44	5,170	4/4	1,000/3,200	12:35	3
0.7	2.47	5,030	5/3	900/2,750	8:07	3
1.0	2.35	4,500	5/3	900/2,750	7:14	3

### MRI Segmentation Approach.

The claustrum was manually segmented in native space by a single rater (SP). Manual segmentation was adopted after testing five recent bespoke automated approaches (16, 20, 24, 85, 101), each of which showed lower consistency with manual segmentations than agreement between human raters, indicating limited generalization (*SI Appendix*, Table S14). Segmentations followed the same protocol as for BigBrain, i.e., were performed in ITK-SNAP using a one-voxel-sized brush, with simultaneous visualization in all three orthogonal planes and three-dimensional rendering, with region-specific preferences (19). Segmentation was based solely on visibility in the MP2RAGE UNI contrast, without reference to the histological gold standard. Given pronounced partial voluming at claustral boundaries, a liberal inclusion criterion was applied, whereby hypointense voxels were included if judged to be predominantly claustral, even when directly abutting adjacent gray matter structures. A second rater (NC) achieved high interrater agreement (average DSC = 0.93, see *SI Appendix*, Fig. S2), and conducted full quality control, described in *SI Appendix*, *Methods* and section 4.

### MRI Alignment and Registration.

Preprocessed MRI scans and native-space segmentations were rigidly aligned to the symmetric MNI ICBM152 nonlinear 2009b template (102), using ANTsRegistration (103) to correct for residual differences in head position that may bias morphometric measurements. All morphometric analyses were performed in this rigidly aligned space. For voxel-wise spatial agreement analyses, rigidly aligned images were subsequently processed using affine (12^°^ of freedom) and symmetric diffeomorphic (SyN) nonlinear registration to the same template, implemented in ANTs (104), with a cross-correlation cost function. The resulting transformations were applied to segmentation labels using GenericLabel interpolation to preserve binary values. Registration accuracy was visually validated by overlaying each subject’s registered anatomy with subcortical structures defined by the Xiao atlas (70). Warped claustrum segmentations were also inspected and showed good alignment, with occasional minor deviations (≤1 voxel) consistent with expected interpolation effects and the thin morphology of the claustrum. To ensure reproducibility, no manual corrections were applied to warped labels.

### Downsampled Histology.

To evaluate the effect of spatial resolution, the gold standard segmentation was downsampled to “MRI-like” isotropic resolutions ranging from 0.4 mm to 2.0 mm (in 0.1 mm increments) across several thresholds (0.2 to 0.8), using FSL’s “flirt” with trilinear interpolation in three dimensions (105, 106). By comparing the downsampled gold standard to segmentations derived from acquired MRI, we effectively test whether spatial resolution alone accounts for observed differences. Substantial discrepancies would suggest that additional factors, such as contrast differences between histological staining and T1-weighted MR imaging, contribute to the difficulty of capturing the claustrum in vivo. An example of downsampling effects is shown in *SI Appendix*, Fig. S8.

### Morphometric Measurements.

We computed six three-dimensional metrics to characterize claustrum segmentations from the histological gold standard, rigidly aligned MRI datasets, and the downsampled gold standards. Volume was calculated as the number of labeled voxels multiplied by voxel resolution, reported in cubic millimeters. Extents were calculated as the maximal dimension along each orthogonal axis (x, y, z), in millimeters. Roundness (dimensionless) was computed as a ratio comparing the surface area of a sphere with the same Feret diameter as the segmentation’s mesh, where values near 1 indicate a spherical shape and lower values reflect increasingly elongated or irregular geometry. Flatness (dimensionless) was calculated as the square root of the ratio between the structure’s second-smallest and smallest eigenvalues, with larger values indicating more planar, sheet-like structures. To complement Extents, we computed the Oriented Bounding Box (OBB), the minimal bounding box (x^′^, y^′^, z^′^) enclosing each claustrum irrespective of axis alignment, in millimeters. OBB was excluded from statistical comparisons to reduce the number of multiple comparisons. All metrics were computed over three-dimensional volumes using the ‘Label Map Statistics’ module in 3D Slicer (v5.6.2) (107). We did not report absolute surface area, as it is ill-defined for structures without a closed surface representation, nor did we normalize metrics by intracranial volume (other than for the analysis of sex differences).

The claustrum’s thin mediolateral profile follows a curved, nonlinear anatomical trajectory; as a result, three-dimensional metrics such as axis-aligned extents and oriented bounding boxes, which integrate across this curvature, can obscure the degree of thinness evident in individual two-dimensional slices. To better capture this property, we computed two two-dimensional (slice-wise) metrics in the coronal plane: “mean thickness, total voxel span” as the distance between the minimum and maximum x-values of segmented voxels in each slice, irrespective of contiguity, and “mean thickness, contiguous voxels” as the average width of all uninterrupted segments along the x-axis, capturing interruptions due to intervening white matter (*SI Appendix*, Fig. S9). These two thickness measures diverge when claustrum segmentation becomes fragmented within individual coronal slices, with the ventral claustrum showing the greatest divergence, measured by the ratio between total span and contiguous thickness. Finally, for visualization, we projected the three-dimensional gold standard segmentation along each orthogonal axis and summed voxel counts to generate flattened thickness maps. (This visualization was not computed for MRI or the downsampled gold standards, as their spatial resolution is insufficient to distinguish anatomical thinness from voxel sampling effects due to partial voluming.)

### Statistical Analysis.

Statistical analyses evaluated resolution-dependent morphometric and spatial agreement effects and explored interindividual and biological variability across MRI datasets; full details are provided in *SI Appendix*, *Methods* and section 5.

## Supplementary Material

Appendix 01 (PDF)

## Data Availability

All newly generated data, including the manually segmented BigBrain-derived claustrum “gold standard,” individual MRI claustrum segmentations in native space (n=30), and the cross-modal probabilistic claustrum atlas, are available via Zenodo at https://doi.org/10.5281/zenodo.19656275 (108). MRI preprocessing code is available at https://github.com/navonacalarco/Claustrum (109). The BigBrain histological dataset (100 μm isotropic resolution) is publicly available at https://ftp.bigbrainproject.org/bigbrain-ftp/ (110). The 0.5 mm isotropic MICA-PNI MRI dataset is publicly available at https://osf.io/mhq3f/overview (111). The 0.7 mm and 1.0 mm isotropic MRI datasets acquired at Maastricht University for prior publications (60, 65) are now made available at https://doi.org/10.5281/zenodo.19656275 (108).
