# Supplementary material for: Multiscale characterization of the human claustrum from histology to MRI
Source: Proc Natl Acad Sci U S A. 2026 Jun 29;123(27):e2604111123. doi: 10.1073/pnas.2604111123 (PMC13342998; doi:10.1073/pnas.2604111123)
Supplement: Supplementary file 1 — Appendix 01 (PDF) [file pnas.2604111123.sapp.pdf]

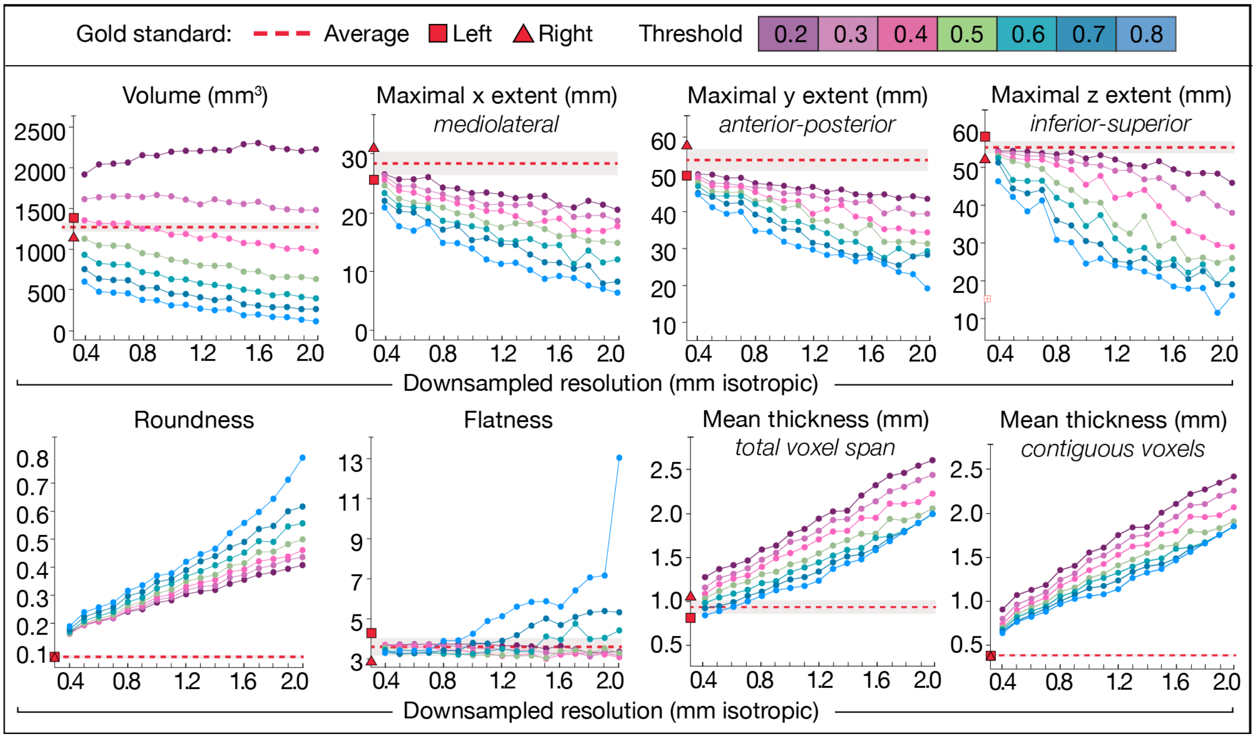

Fig. S1. Downsampling of the histological gold standard to various MRI-like resolutions, at different thresholds. Downsampled estimates of the eight morphometric measurements, averaged across hemispheres, to resolutions of 0.4-2.0mm. The gold standard's measurements are shown in red (dashed line = average, square = left hemisphere, triangle = right hemisphere). Each coloured line represents a different binarisation threshold (0.2-0.8).

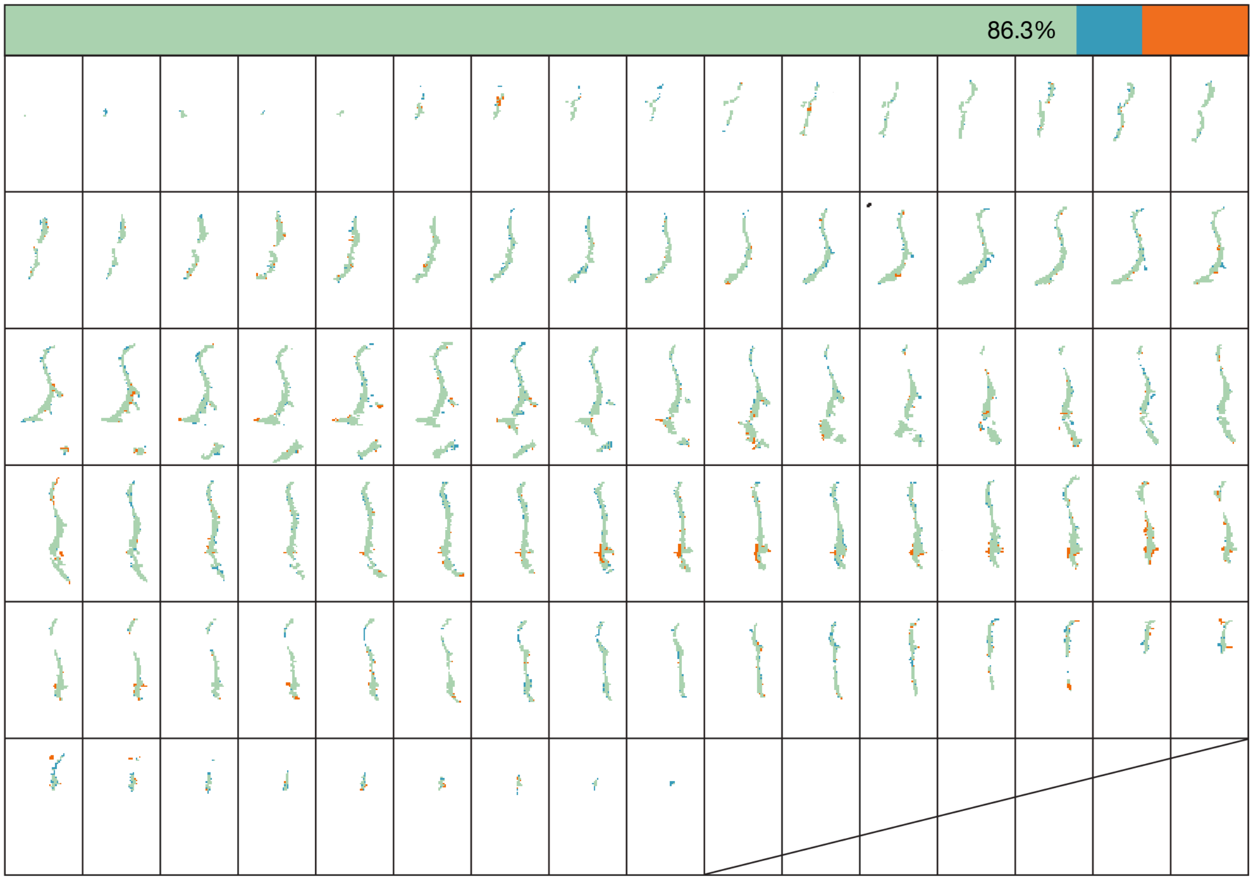

Fig. S2. Inter-rater agreement of MRI segmentation. The left hemisphere from the participant with the most average volume in each dataset was segmented independently by two raters. Agreement assessed using Dice Similarity Coefficient (DSC) showed high structural overlap at all resolutions: DSC=0.926 at 0.5mm, 0.941 at 0.7mm, and 0.934 at 1.0mm isotropic. Coronal slices (anterior-to-posterior) show left hemisphere segmentations from both raters for the 0.5mm dataset participant with lowest agreement (DSC=0.926). Rater 1 (SP, orange), Rater 2 (NC, blue), and overlap (green); voxels segmented by only one rater shown in their respective color. Horizontal bar shows proportions of agreement (86.3%) and disagreement (13.7%). Consistent with gold standard segmentation (**Extended Data Fig. 2**), disagreements occurred primarily along edges and in the ventral claustrum.

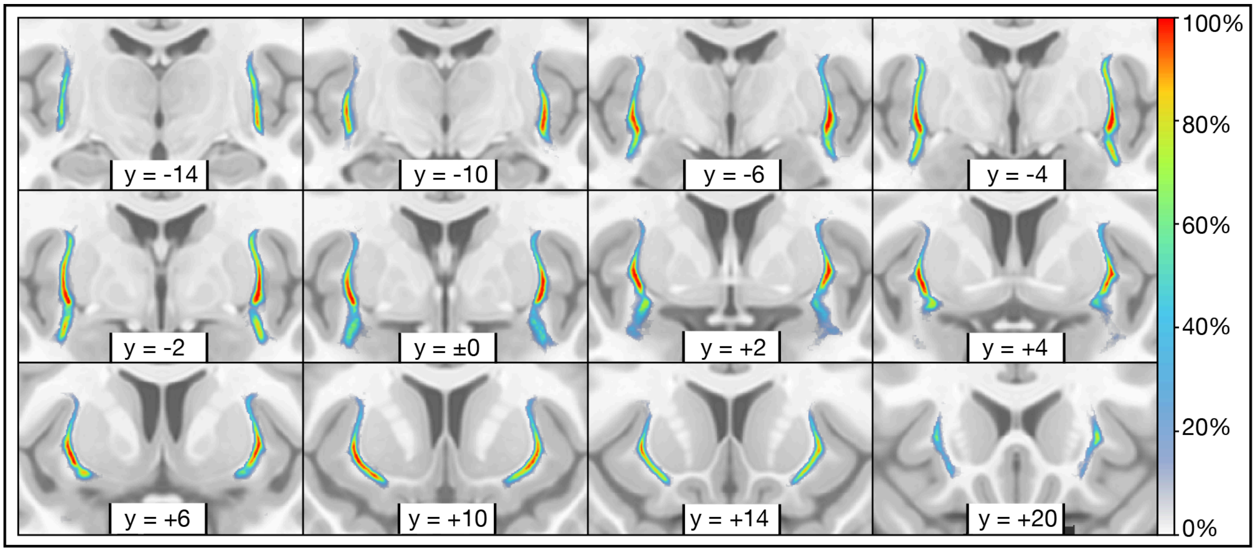

Fig. S3. Probabilistic overlay of MRI datasets. Coronal slices show voxel-wise overlap of MNI-aligned claustrum segmentations from all three MRI datasets (n=30), resampled to 0.5mm isotropic resolution. Voxel intensity reflects the proportion of participants with claustrum present at each location (0–100%). A consistent central core spans the anteroposterior extent (Y=-14 to +20mm shown), with highest agreement in the dorsal midsection. Variability increases toward the periphery, particularly ventrally and anteriorly, reflecting reduced thickness and greater boundary ambiguity.

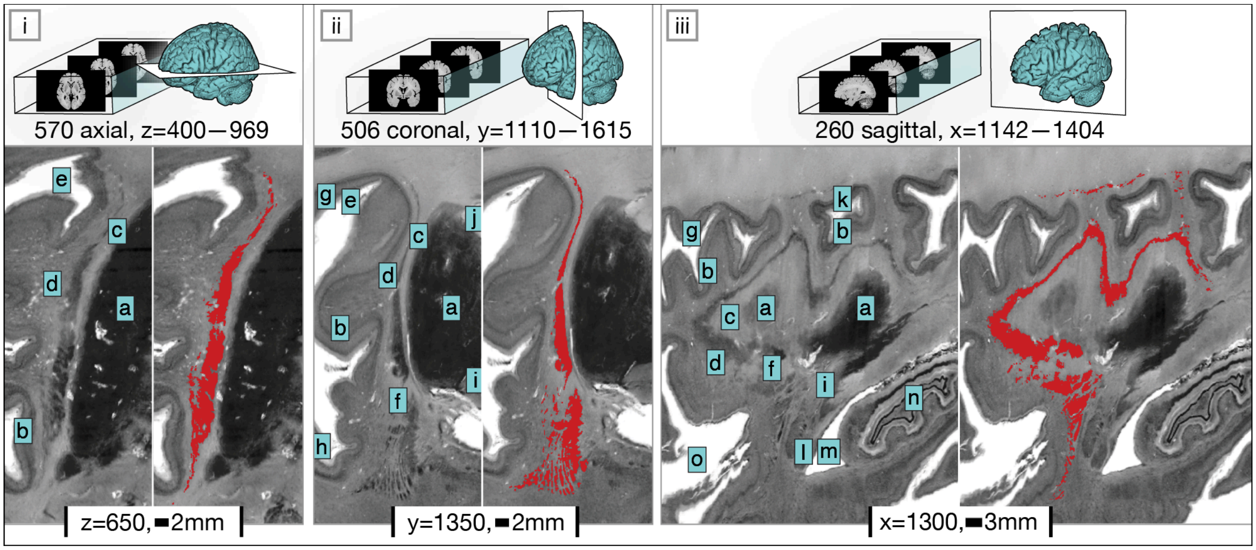

Fig. S4. **BigBrain-derived gold standard claustrum model**. The right hemisphere claustrum delineation is shown, with vertical panels corresponding to approximately midpoint (i) axial, (ii) coronal, and (iii) sagittal views. The top row indicates the number of slices with a claustrum label, and visualises the location of the slice shown below (BigBrain coordinate given). The bottom row displays cropped BigBrain (left) alongside the corresponding claustrum label in red (right), highlighting the extraordinary detail achieved via slice-wise manual segmentation with a one-voxel brush. Letters mark nearby structures and spaces: (a) putamen, (b) insular cortex, (c) external capsule, (d) extreme capsule, (e) circular sulcus, (f) uncinate fascicle, (g) frontal operculum, (h) planum temporale, (i) anterior commissure, (j) internal capsule, (k) parietal operculum, (l) lateral amygdaloid nucleus, (m) lateral ventricle, (n) hippocampus, (o) lateral sulcus.

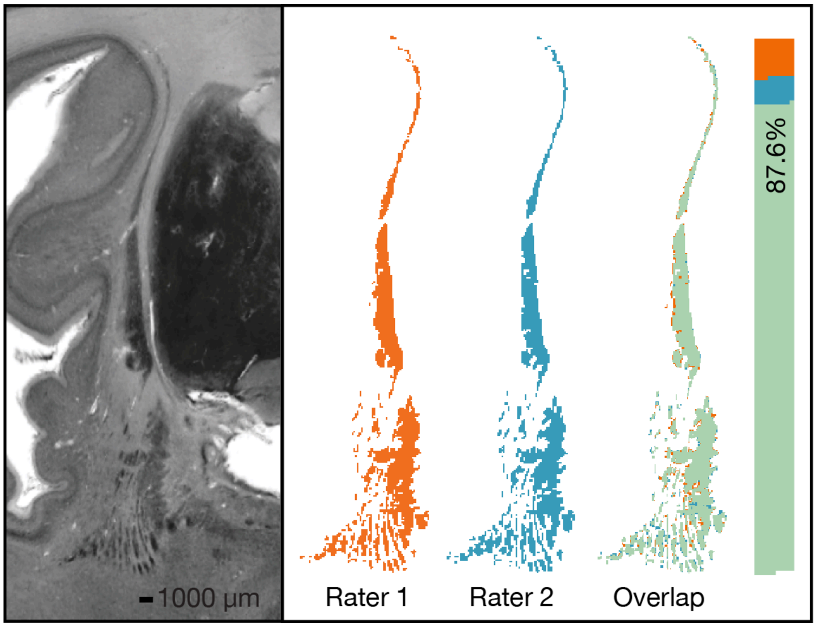

Fig. S5. Inter-rater agreement of gold standard claustrum segmentation. Inter-rater agreement was assessed via duplicate segmentation of three randomly selected coronal slices in the right hemisphere, spaced ≥75 slices apart and containing >100 voxels in both segmentations. Rater 2 segmented *de novo* without access to Rater 1's work. Dice Similarity Coefficient (DSC) ranged from 0.87 to 0.93, indicating high inter-rater agreement. Best-case agreement shown for a single slice (BigBrain y=1350, DSC=0.93). Left: BigBrain histology. Right: Segmentations by Rater 1 (SP, orange, 6100 voxels) and Rater 2 (NC, blue, 5960 voxels), with overlap (green) and unique voxels in respective colors. Bar shows agreement (87.6%) and disagreement (12.4%). Disagreements primarily occurred along edges and in ventral "puddles."

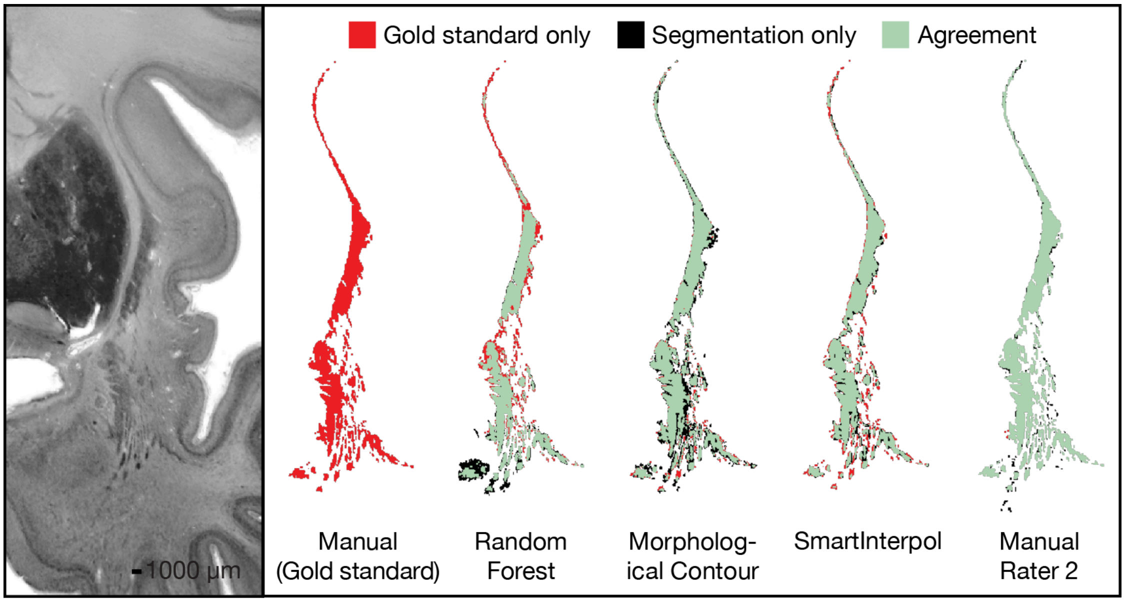

Method	DSC, all 10 segmentations (mean, sd)	DSC, segmentation y=1348
Morphological contour	0.85 (0.02)	0.83
Random forest	0.83 (0.02)	0.81
SmartInterpol	0.84 (0.02)	0.85

Fig. S6. Testing 3 automated segmentation algorithms on histology.
We tested three sparse interpolation algorithms with default parameters to evaluate their potential for reducing manual segmentation workload: morphological contour (1), random forest (2), and SmartInterpol (3) (using the product rule segmentation, which combines label fusion and deep learning). In a test region encompassing the ventral claustrum as it extends into the temporal lobe (28 consecutive coronal slices, BigBrain coordinates y=1335-1365), we manually segmented all slices but provided only every third slice (including the first and last) to each algorithm. On the task of segmenting interleaved 10 segmentations, all three methods produced good agreement with manual segmentation (see Table, below). In contrast, two human raters achieved excellent agreement (DSC=0.97) on a test slice (y=1348) on which all algorithms showed just good agreement. Lower algorithmic performance may stem from the claustrum's highly undulating morphology between slices, violating the algorithms' assumptions of high inter-slice correlation. Certainly, all methods would likely show improved results with tuning, but for challenging regions like the ventral claustrum, we judged that manual segmentation was essential and remains best practice. The higher human inter-rater agreement observed here (compared to that reported in **Extended Data Fig. 2**) may be because Rater 2 was provided with the same sparse input as the algorithms; in the earlier comparison, segmentation was performed *de novo*.

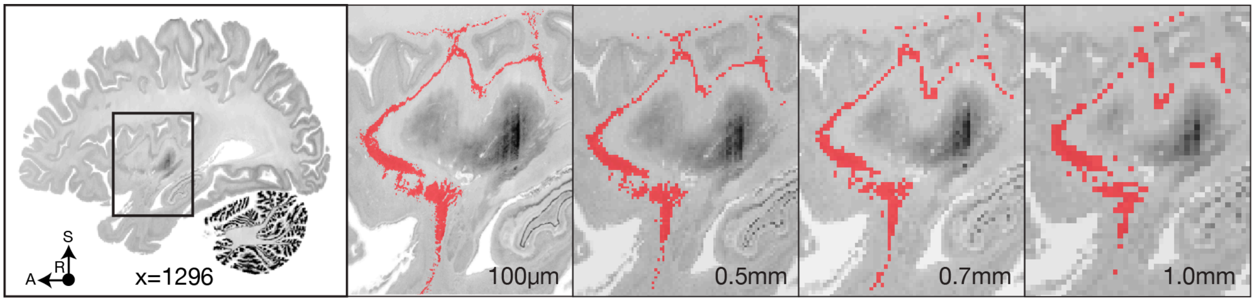

Fig. S7. Downsampling analysis. Left: Inset shows a sagittal view of the BigBrain dataset (slice x=1296) in the right hemisphere, with a box indicating the zoomed region shown in subsequent panels. Right: The first panel displays the gold standard claustrum segmentation at 100µm resolution (red), followed by the same segmentation after downsampling to resolutions matched to the three acquired MRI datasets, thresholded at 50%. The comparison illustrates how spatial resolution affects anatomical detail: while gross shape and topology are preserved at submillimetric levels, finer features are progressively lost at lower resolutions.

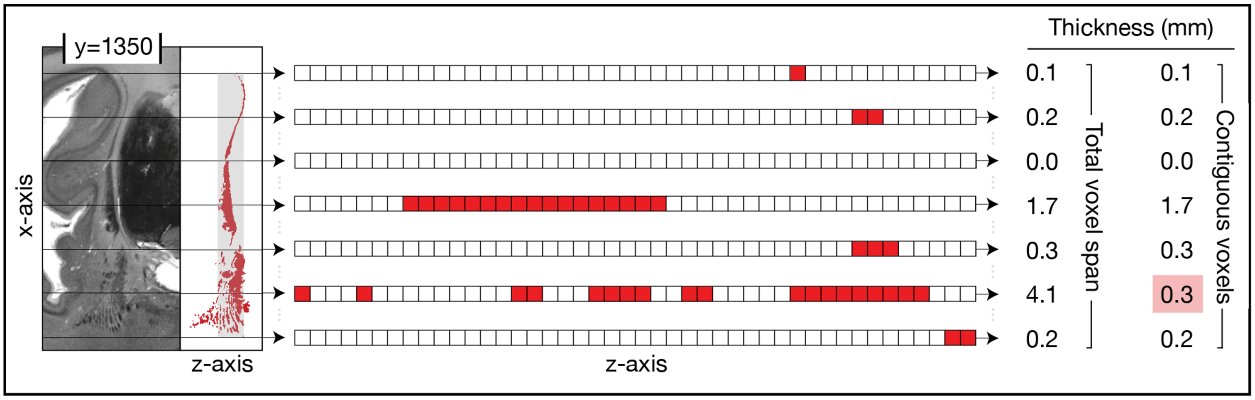

Fig. S8. Slice-wise calculations to capture claustral thinness. Two slice-wise metrics were computed across the anteroposterior extent to quantify claustral thinness. The illustration shows how `mean thickness, total voxel span`, and `mean thickness, contiguous voxels` were calculated for an example coronal slice (y=1350) of the right hemisphere of the gold standard, near the claustrum's midpoint. Left: the histological image and corresponding segmentation (red) illustrate variation in claustral thickness in two dimensions along the x-axis. This variability is further compounded in three dimensions, as the claustrum follows a curved trajectory from anterior to posterior. Middle: seven equidistant positions along the x-axis (of 455 total) are highlighted. Right: the table shows counts for both metrics, and highlights (pink) differences in the ventral claustrum. Mean thickness of contiguous voxels, which adjusts for white matter interruptions, is particularly relevant for MRI where partial voluming may cause ventral "puddles" to fall below detection thresholds or appear artefactually thickened. In the slice shown, the mean total voxel span was 2.46mm, while the mean thickness of contiguous voxels was 1.16mm (ratio=2.12).

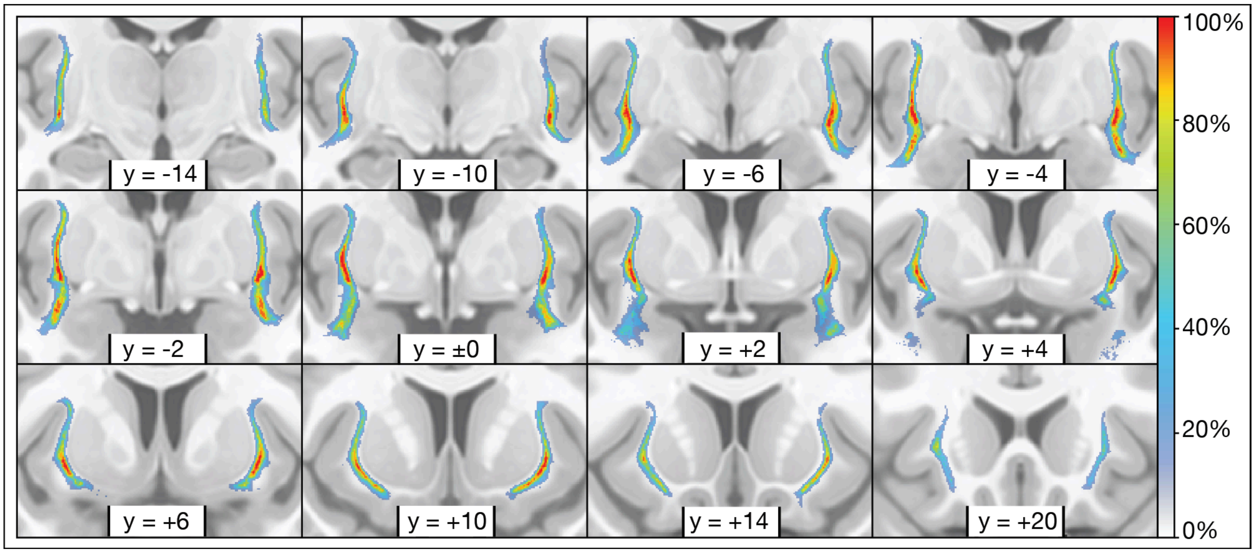

Fig. S9. Cross-modal high-resolution probabilistic claustrum atlas. A voxelwise probabilistic atlas was constructed by integrating the histological gold standard (BigBrain, 100μm), manual claustrum segmentations from the 0.5mm 7-Tesla MRI dataset (n = 10), and two previously released high-resolution MRI segmentations from Mauri and colleagues (4): one *ex vivo* at 100μm (5) and one *in vivo* at 250μm (6) (see **Table S15**). All segmentations were aligned to symmetric MNI ICBM152 nonlinear 2009b space, resampled to 0.5mm isotropic resolution, and combined using differential weighting favouring higher-resolution datasets (see **SI Methods, section 2**). Coronal slices are shown across the anterior–posterior extent (y=−14 to +20). Voxel intensity reflects the weighted likelihood of claustral tissue at each location (0–100%). Highest spatial agreement is observed in the dorsal mid-claustrum, with increasing variability toward the superior, anterior, and ventral periphery, consistent with partial-voluming and individual variability. The probabilistic atlas provides a cross-modal anatomical prior for claustrum localisation while explicitly encoding boundary uncertainty.

	Gold standard	Downsampled gold standards			MRI datasets		
**Resolution**	**100μm**	**0.5mm**	**0.7mm**	**1.0mm**	**0.5mm**	**0.7mm**	**1.0mm**
Three-dimensional							
**Volume (mm^3)**	1268.01 (81.42)	1042.12 (101.65)	1030.54 (106.96)	864.00 (77.78)	1459.14 (183.20)	1371.33 (126.61)	1318.80 (400.38)
**Maximal x extent (mm)** (mediolateral)	28.35 (2.90)	22.00 (2.12)	21.00 (1.98)	19.00 (1.41)	18.65 (1.53)	16.91 (1.23)	15.35 (1.95)
**Maximal y extent (mm)** (anteroposterior)	53.45 (4.03)	45.00 (0.71)	44.80 (0.99)	40.50 (0.71)	47.83 (3.46)	45.71 (3.37)	39.75 (4.40)
**Maximal z extent (mm)** (inferosuperior)	55.45 (2.19)	51.75 (0.35)	49.70 (0.99)	41.00 (0.00)	36.42 (2.25)	35.49 (3.74)	33.00 (2.38)
**OBB x’**	24.41 (2.30)	18.58 (2.55)	17.89 (2.22)	16.71 (0.15)	15.74 (1.42)	14.52 (1.24)	11.96 (1.37)
**OBB y’**	47.35 (0.49)	45.30 (0.11)	45.05 (0.90)	36.56 (3.65)	36.59 (1.94)	34.85 (3.59)	32.83 (2.02)
**OBB z’**	57.26 (1.62)	50.35 (0.73)	50.19 (0.93)	44.50 (5.10)	48.16 (3.18)	46.61 (3.01)	41.10 (4.24)
**Roundness**	0.08 (0.00)	0.20 (0.00)	0.23 (0.00)	0.31 (0.01)	0.23 (0.01)	0.27 (0.01)	0.35 (0.02)
**Flatness**	3.58 (0.62)	3.46 (0.72)	3.39 (0.72)	3.17 (0.75)	3.51 (0.43)	3.46 (0.33)	3.80 (0.45)
Two-dimensional							
**Mean thickness, total voxel span (mm)**	0.97 (0.60)	1.15 (0.48)	1.25 (0.46)	1.44 (0.34)	1.69 (0.50)	1.75 (0.51)	2.14 (0.49)
**Mean thickness, contiguous voxels (mm)**	0.48 (0.17)	0.97 (0.32)	1.10 (0.32)	1.39 (0.31)	1.61 (0.43)	1.72 (0.49)	2.14 (0.49)

Table S1. Morphological metrics for the gold standard, the gold standard downsampled to three acquired MRI resolutions (thresholded at 50%), and MRI datasets. Values are averaged across hemispheres. For the gold standard and downsampled gold standards (one brain), bracketed values indicate inter-hemispheric differences and should not be interpreted as a true standard deviation. For MRI datasets (each n=10), bracketed values indicate standard deviation. See **Table S2 and S3** for hemisphere-specific results.

Left hemisphere

	Gold standard	Gold standard downsampled			MRI datasets		
**Resolution**	**100μm**	**0.5mm**	**0.7mm**	**1.0mm**	**0.5mm**	**0.7mm**	**1.0mm**
Three-dimensional							
**Volume (mm^3)**	1325.58	1114	1106.17	919	1408.26 (186.99)	1329.23 (108.04)	1274.6 (367.39)
**Maximal x extent (mm)** (mediolateral)	26.30	20.50	19.60	18.00	18.50 (1.58)	16.87 (1.30)	15.00 (1.70)
**Maximal y extent (mm)** (anteroposterior)	56.30	44.50	44.10	40.00	47.60 (3.96)	44.87 (3.19)	40.00 (5.16)
**Maximal z extent (mm)** (inferosuperior)	53.90	51.50	49.00	41.00	36.70 (2.41)	35.28 (3.71)	33.00 (3.02)
**OBB x' (mm)**	22.79	16.78	16.32	16.60	15.18 (1.51)	14.32 (1.03)	12.03 (1.06)
**OBB y' (mm)**	47.00	45.23	44.41	39.14	36.26 (2.14)	34.25 (3.66)	32.53 (2.38)
**OBB z' (mm)**	58.41	49.84	49.53	40.90	47.72 (3.69)	45.72 (2.33)	41.03 (5.17)
**Roundness**	0.08	0.20	0.23	0.30	0.23 (0.01)	0.27 (0.01)	0.36 (0.02)
**Flatness**	4.02	3.97	3.90	3.70	3.45 (0.48)	3.37 (0.34)	3.63 (0.25)
Two-dimensional							
**Mean thickness, total voxel span (mm)**	0.91 (0.61)	1.16 (0.48)	1.26 (0.45)	1.43 (0.34)	1.70 (0.49)	1.73 (0.47)	2.15 (0.44)
**Mean thickness, contiguous voxels (mm)**	0.48 (0.20)	0.98 (0.32)	1.13 (0.32)	1.38 (0.30)	1.60 (0.41)	1.71 (0.46)	2.15 (0.44)

Table S2. Morphological metrics for the left claustrum. Conventions are as in Table S1.

Right hemisphere

	Gold standard	Gold standard downsampled			MRI datasets		
**Resolution**	**100μm**	**0.5mm**	**0.7mm**	**1.0mm**	**0.5mm**	**0.7mm**	**1.0mm**
Three-dimensional							
**Volume (mm^3)**	1210.44	970.25	954.91	809	1510.03 (173.59)	1413.43 (135.03)	1363.00 (446.21)
**Maximal x extent (mm)** (mediolateral)	30.40	23.50	22.40	20.00	18.80 (1.55)	16.94 (1.23)	15.70 (2.21)
**Maximal y extent (mm)** (anteroposterior)	50.60	45.50	45.50	41.00	48.05 (3.07)	46.55 (3.50)	39.50 (3.75)
**Maximal z extent (mm)** (inferosuperior)	57.00	52.00	50.40	41.00	36.15 (2.17)	35.70 (3.96)	33.00 (1.70)
**OBB x' (mm)**	22.79	20.39	19.47	16.82	16.29 (1.15)	14.73 (1.45)	11.90 (1.68)
**OBB y' (mm)**	47.69	45.38	45.68	33.98	36.91 (1.76)	35.45 (3.61)	33.12 (1.67)
**OBB z' (mm)**	56.12	50.87	50.85	48.10	47.72 (48.60)	47.50 (3.46)	41.16 (3.36)
**Roundness**	0.08	0.20	0.23	0.32	0.22 (0.01)	0.27 (0.01)	0.35 (0.02)
**Flatness**	3.15	2.96	2.88	2.64	3.58 (0.39)	3.55 (0.30)	3.97 (0.55)
Two-dimensional							
**Mean thickness, total voxel span (mm)**	1.04 (0.60)	1.14 (0.49)	1.25 (0.46)	1.46 (0.34)	1.68 (0.50)	1.77 (0.54)	2.13 (0.54)
**Mean thickness, contiguous voxels (mm)**	0.48 (0.15)	0.95 (0.32)	1.08 (0.31)	1.41 (0.31)	1.61 (0.45)	1.73 (0.52)	2.13 (0.54)

Table S3. Morphological metrics for the right claustrum. Conventions are as in Table S1.

		Left hemisphere coordinates (mm)			Right hemisphere coordinates (mm)		
**Dataset type**	**Dataset resolution (mm isotropic)**	**x**	**y**	**z**	**x**	**y**	**z**
BigBrain	0.1	-32.32	+0.90	-5.37	31.68	1.09	-6.01
MRI	0.5	-32.45 (0.53)	1.43 (0.88)	-3.03 (1.81)	32.09 (0.19)	2.38 (0.78)	-3.57 (1.93)
MRI	0.7	-32.56 (0.28)	0.83 (0.84)	-2.67 (1.82)	32.14 (0.24)	1.90 (1.10)	-4.04 (1.86)
MRI	1.0	-33.26 (0.62)	-0.24 (1.24)	-2.35 (1.78)	31.99 (0.46)	1.49 (1.30)	-3.69 (1.82)

Table S4. MNI coordinates of claustrum centre of mass. Centre of mass coordinates (x, y, z) for left and right claustra across the gold standard and MRI datasets, in MNI space (mm). MRI-derived centres closely approximate the gold standard, with most falling within several voxel's distance.

	Corrected p value	η^2	Significant pairwise differences (p value)
Three-dimensional			
**Volume (mm^3)**	0.246	0.048	*None*
**Maximal x extent (mm)** (mediolateral)	<0.001	0.428	0.5mm vs 0.7mm = 0.003<br>0.5m vs 1.0mm < 0.001<br>0.7mm vs 1.0mm = 0.009
**Maximal y extent (mm)** (anteroposterior)	<0.001	0.464	0.5mm vs 1.0mm < 0.001<br>0.7mm vs 1.0mm < 0.001
**Maximal z extent (mm)** (inferosuperior)	0.002	0.211	0.5mm vs 1.0mm = 0.001<br>0.7mm vs 1.0mm = 0.022
**Roundness**	<0.001	0.945	0.5mm vs 0.7mm < 0.001<br>0.5mm vs 1.0mm < 0.001<br>0.7mm vs 1.0mm < 0.001
**Flatness**	0.025	0.126	0.7mm vs 1.0mm = 0.027
Two-dimensional			
**Mean thickness, total voxel span (mm)**	<0.001	0.691	0.5mm vs 1.0mm = < 0.001<br>0.7mm vs 1.0mm = < 0.001
**Mean thickness, contiguous voxels (mm)**	<0.001	0.758	0.5mm vs 0.7mm = 0.026<br>0.5mm vs 1.0mm < 0.001<br>0.7mm vs 1.0mm < 0.001

Table S5. Statistical comparison of the MRI datasets on all morphometric measurements.

Resolution (mm)	0.5mm	0.7mm	1.0mm
Three-dimensional			
**Volume (mm^3)**	0.12	0.08	0.31
**Maximal x extent (mm)** (mediolateral)	0.06	0.06	0.10
**Maximal y extent (mm)** (anteroposterior)	0.06	0.10	0.05
**Maximal z extent (mm)** (inferosuperior)	0.06	0.06	0.11
**Roundness**	0.04	0.04	0.06
**Flatness**	0.10	0.08	0.08
Two-dimensional			
**Mean thickness, total voxel span (mm)**	0.07	0.07	0.07
**Mean thickness, contiguous voxels (mm)**	0.06	0.07	0.07

Table S6. Coefficient of variation (CV) of morphometric measurements in MRI datasets. Variability across participants within each MRI dataset. All metrics showed low variability (CV<0.15) except volume at 1.0 mm (CV=0.31), indicating reduced measurement stability at lower resolution.

	Gold standard vs MRI datasets					
	Percent difference			Corrected p value		
**Resolution (mm)**	**0.5**	**0.7**	**1.0**	**0.5**	**0.7**	**1.0**
Three dimensional						
**Volume (mm^3)**	+15.30	+8.36	+4.26	<0.001	0.004	0.608
**Maximal x extent (mm)** (mediolateral)	-33.72	-40.03	-45.58	<0.001	<0.001	<0.001
**Maximal y extent (mm)** (anteroposterior)	-10.25	-14.15	-25.45	<0.001	<0.001	<0.001
**Maximal z extent (mm)** (inferosuperior)	-34.29	-35.97	-40.45	<0.001	<0.001	<0.001
**Roundness**	+186.05	+242.40	+344.79	<0.001	<0.001	<0.001
**Flatness**	-0.57	-1.97	+7.07	0.608	0.360	0.063
Two-dimensional						
**Mean thickness, total voxel span (mm)**	+74.30	+80.03	+120.81	<0.001	<0.001	<0.001
**Mean thickness, contiguous voxels (mm)**	+234.3	+257.41	+343.74	<0.001	<0.001	<0.001

Table S7. Differences between gold standard and MRI datasets. Positive percent differences indicate MRI values exceed gold standard values. All p-values were corrected using false discovery rate (FDR) correction across 24 comparisons.

	Gold standard	Gold standard downsampled			MRI datasets		
**Resolution**	**100μm**	**0.5mm**	**0.7mm**	**1.0mm**	**0.5mm**	**0.7mm**	**1.0mm**
**Span-to-contiguous thickness ratio: full extent**	1.85 (0.79)	1.16 (0.20)	1.11 (0.17)	1.04 (0.09)	1.05 (0.10)	1.01 (0.05)	1.00 (0.01)
**Span-to-contiguous thickness ratio: middle third**	2.76 (0.58)	1.35 (0.21)	1.28 (0.20)	1.08 (0.13)	1.08 (0.10)	1.02 (0.04)	1.00 (0.01)
**Maximum ratio: full extent**	4.29	1.86	1.69	1.43	2.40	1.68	1.20

Table S8. Ratio between total voxel span and contiguous thickness for each dataset, computed across the full claustrum and within the middle third of the anteroposterior axis. The gold standard shows large discrepancies, whereas MRI ratios approach 1.00, reflecting resolution-driven loss of anatomical detail.

	Gold standard vs. downsampled gold standard			Gold standard vs. MRI			Downsampled gold standard vs. MRI		
	**0.5**	**0.7**	**1.0**	**0.5**	**0.7**	**1.0**	**0.5**	**0.7**	**1.0**
**DSC**	0.82 (0.02)	0.81 (0.02)	0.71 (0.02)	0.39 (0.02)	0.40 (0.03)	0.37 (0.03)	0.41 (0.03)	0.44 (0.03)	0.41 (0.04)
**HD (mm)**	6.28 (0.23)	6.80 (2.34)	13.28 (0.67)	9.49 (2.35)	12.12 (2.99)	13.05 (2.23)	8.29 (1.55)	9.98 (3.10)	10.65 (1.78)
**dDSC**	0.82 (0.02)	0.83 (0.03)	0.70 (0.03)	0.59 (0.02)	0.63 (0.02)	0.61 (0.03)	0.60 (0.02)	0.66 (0.03)	0.64 (0.05)
**baHD (mm)**	0.16 (0.02)	0.22 (0.05)	0.69 (0.08)	0.71 (0.07)	0.83 (0.16)	1.18 (0.13)	0.71 (0.09)	0.75 (0.15)	1.15 (0.29)
**Jaccard (IoU)**	0.69 (0.02)	0.68 (0.02)	0.55 (0.02)	0.24 (0.02)	0.26 (0.02)	0.23 (0.02)	0.26 (0.02)	0.28 (0.02)	0.26 (0.03)

Table S9. Agreement between gold standard, downsampled gold standard, and MRI datasets.

	Downsampled gold standards vs MRI datasets					
	Percent difference			Corrected p value		
**Resolution (mm)**	**0.5**	**0.7**	**1.0**	**0.5**	**0.7**	**1.0**
Three-dimensional						
**Volume (mm^3)**	+40.66	+33.76	+53.34	<0.001	<0.001	<0.001
**Maximal x extent (mm)** (mediolateral)	-14.64	-19.11	-19.00	<0.001	<0.001	<0.001
**Maximal y extent (mm)** (anteroposterior)	+6.29	+2.03	-1.83	0.002	0.260	0.504
**Maximal z extent (mm)** (inferosuperior)	-29.62	-28.59	-19.51	<0.001	<0.001	<0.001
**Roundness**	+13.83	+18.19	+15.27	<0.001	<0.001	<0.001
**Flatness**	+3.53	+4.47	+22.42	0.731	0.624	<0.001
Two-dimensional						
**Mean thickness, total voxel span (mm)**	+47.27	+39.56	+48.21	<0.001	<0.001	<0.001
**Mean thickness, contiguous voxels (mm)**	+66.61	+55.98	+53.49	<0.001	<0.001	<0.001

Table S10. Differences between resolution-matched downsampled gold standards (50% threshold) and MRI. Positive percent differences indicate MRI values exceed downsampled gold standard values. All p-values were corrected using false discovery rate (FDR) correction across 24 comparisons.

	Downsampled gold standard vs. MRI		
**Resolution (mm)**	**0.5**	**0.7**	**1.0**
**DSC efficiency (%)**	50.00	54.32	57.75
**HD efficiency (%)**	75.75	68.14	124.69

Table S11. MRI performance efficiency relative to theoretical limits. Dice similarity coefficient (DSC) and Hausdorff distance (HD) efficiency for each MRI dataset, defined as the proportion of achievable volumetric overlap or boundary precision recovered relative to the theoretical ceiling (downsampled vs. gold standard). See also **Fig. 4C**.

		Edlow MGH dataset (2019)	
	Gold standard	Coates & Zaretskaya (2024)	Mauri and colleagues (2025)
	**100µm**	**100µm**	**100µm**
Three-dimensional			
**Volume (mm^3)**	1268.01 (81.42)	1905.32 (239.12)	1453.46 (21.78)
**Maximal x extent (mm)** (mediolateral)	28.35 (2.90)	25.65 (4.31)	19.95 (0.21)
**Maximal y extent (mm)** (anteroposterior)	53.45 (4.03)	53.70 (7.50)	45.10 (1.41)
**Maximal z extent (mm)** (inferosuperior)	55.45 (2.19)	51.35 (1.20)	40.85 (3.32)
**OBB x'**	24.41 (2.30)	20.59 (4.78)	15.33 (1.05)
**OBB y'**	47.35 (0.49)	53.72 (1.64)	43.18 (3.40)
**OBB z'**	57.26 (1.62)	53.39 (5.01)	46.45 (1.00)
**Roundness**	0.08 (0.00)	0.16 (0.05)	0.17 (0.02)
**Flatness**	3.58 (0.62)	4.40 (0.79)	4.48 (0.56)
Two-dimensional			
**Mean thickness, total voxel span (mm)**	0.97 (0.60)	1.16 (0.64)	1.14 (0.56)
**Mean thickness, contiguous voxels (mm)**	0.48 (0.17)	1.07 (0.52)	1.09 (0.50)
**Span-to-contiguous thickness ratio: full extent**	1.85 (0.79)	1.08 (0.21)	1.04 (0.08)
**Span-to-contiguous thickness ratio: middle third**	2.76 (0.58)	1.20 (0.31)	1.10 (0.10)

Table S12. Claustrum morphometrics for super-high resolution *ex vivo* MRI (100µm; single brain) (5), independently segmented by Coates & Zaretskaya (7) and Mauri and colleagues (4).

Gold standard values are included for comparison. Values reflect the average across hemispheres; bracketed values reflect inter-hemispheric differences, not standard deviations.

Dataset resolution (mm isotropic)	Scanner location	N	Sex (female)	Age (mean, SD)
0.5	Montreal	10	6	26.60 (4.60)
0.7	Maastricht	10	4	28.60 (4.17)
1.0	Maastricht	10	5	25.70 (2.94)

Table S13. Demographic details of three MRI datasets (8–10).

Algorithm	DSC	HD	dDSC	baHD
Albishri [2022] (11)	0.21 (0.17)	88.12 (14.94)	0.26 (0.19)	48.24 (8.36)
Berman [2020] (12)	0.42 (0.10)	30.67 (4.45)	0.46 (0.09)	21.59 (3.22)
Brun [2022] (13)	0.69 (0.02)	16.48 (3.96)	0.74 (0.03)	11.75 (2.44)
Li [2021] (14)	0.47 (0.10)	80.31 (21.22)	0.50 (0.10)	39.79 (7.26)
Mauri [2025] (4)	0.62 (0.02)	13.59 (1.70)	0.72 (0.02)	11.29 (1.39)

Table S14. Testing of automated claustrum segmentation algorithms. Manual claustrum segmentations of the 0.5mm dataset compared to five automated algorithms for adult brains (4, 11–14). Agreement was assessed using Dice Similarity Coefficient (DSC), Hausdorff Distance (HD), dilated DSC (dDSC), and balanced average HD (baHD). Values are mean (SD) across n=10 participants. Brun and Mauri's algorithms were developed for 7-Tesla; others 3T. All algorithms except Mauri's were trained on lower resolution data than that to which we applied them here (Brun=0.6mm, Berman=0.7mm, Albishri=0.7mm, and Li=1.0mm, all isotropic voxels). Note that Berman's method is designed for dorsal claustrum only.

Dataset resolution (mm isotropic)	Dataset	N	Segmentation provided by	Modality	Acquisition	Total Weight
0.10	BigBrain (15)	1	This paper	Histology	*Ex vivo*	20%
0.10	Edlow MGH brain (5)	1	Mauri [2025] (4)	7T MRI	*Ex vivo*	20%
0.25	Lüsebrink brain (6)	1	Mauri [2025] (4)	7T MRI	*In vivo*	20%
0.50	0.5mm dataset (9)	10	This paper	7T MRI	*In vivo*	40%

Table S15. Datasets used in creation of cross-modal, probabilistic claustrum atlas.

SI Discussion

Section 1: Suggestions for reporting.

The present findings highlight several factors that substantially influence claustrum measurement and interpretation in MRI. To improve transparency and comparability across studies, we outline practical considerations that may be useful when reporting claustrum analyses:

1. **Spatial resolution.** Report nominal voxel size and effective resolution at the capsule–claustrum boundary in the mediolateral direction, and interpret these relative to the histological gold standard's mean contiguous thickness (~0.56mm).
2. **Acquisition geometry.** Specify the slice plane and its obliquity relative to AC–PC, and to an insula-aligned oblique-coronal plane parallel to the extreme and external capsules.
3. **Image contrast.** Report claustrum-to-capsular contrast-to-noise ratio (CNR).
4. **Segmentation protocol.** Describe the segmentation approach (manual or semi-automatic), any initialisation strategies (e.g., use of an atlas), inter- and intra-rater reliability, and any post hoc topology corrections.
5. **Registration and space.** Where possible, perform segmentation in native space. For group analyses, describe the non-linear registration approach and any local refinement near the claustrum.
6. **Resolution-dependent visibility.** State explicitly which features visible in histology are not detectable in MRI; if some participants were differentially affected (e.g., with ventral "drop out"), consider exclusion criteria based on per-subject claustral capture, though this risks non-random missingness.
7. **Morphometry.** Report morphometric measures beyond volume; we recommend the eight two-dimensional and three-dimensional metrics used here.

SI Methods

Section 1: Creation of cross-modal, probabilistic claustrum atlas.

The atlas comprises segmentations from 13 brains; dataset details are provided in **Table S15**, the atlas is visualised in **Fig. S9**, and it is publicly available at https://github.com/navonacalarco/claustrum. To generate the atlas in standard space, all claustrum segmentations were aligned to the MNI ICBM152 nonlinear 2009b template (16) at 0.5mm isotropic resolution. For the two *ex vivo* datasets, publicly available MNI-aligned versions (BigBrain: https://osf.io/xkqb3/overview; Edlow MGH brain: https://datadryad.org/dataset/doi:10.5061/dryad.119f80q) were used as registration references as they provided superior subcortical alignment. Both *in vivo* datasets were registered directly to the MNI template, following the procedures outlined in the ‘Non-linear registration’ subsection of **SI Methods, section 4**. Differential weighting was applied such that higher-resolution datasets exerted greater influence on the final voxelwise probabilities.

Section 2: Manual claustrum localisation and segmentation in BigBrain

Localization. To enable real-time navigation of the massive BigBrain dataset (dimensions: x=1970, y=2330, z=1890), we extracted smaller volumes for each hemisphere encompassing the claustrum and its surrounding structures (dimensions: x_{left} =400-900; x_{right}=1000-1500, y=1000-1775, z=400-1000). References to the left and right claustra follow neurological convention.

Segmentation. Current understanding of human claustrum anatomy is informed by anatomical studies (17–20) and whole-brain histological atlases (21, 22). However, given considerable discordance in boundary illustrations across sources, our delineation prioritised apparent voxel intensity in BigBrain, reflecting the presence of neurons (cell bodies) amongst brighter surroundings (white matter). This criterion permits isolated voxels to be labelled as claustral in principle; however, in practice, nearly all included voxels were contiguous in at least one anatomical plane. We treated the claustrum as a single unified structure, as the number, location, and nomenclature of proposed claustral subsections have been debated for more than a century (23) and even contemporary atlases using similar methods depict markedly different subdivisions (21, 22). In practice, most researchers treat the claustrum as one, using "dorsal" and "ventral" to refer to positions along the superior-inferior axis where morphology markedly differs. Consistent with prevailing practice, we therefore use the terms "dorsal" and "ventral" descriptively to denote relative positions along the superior–inferior axis, rather than to imply discrete anatomical subdivisions.

Automated segmentation. Before proceeding with full manual segmentation, we evaluated several interpolation-based segmentation approaches, namely morphological contour (1), random forest (2), and SmartInterpol (3), using default parameters. Methods were tested on 28 consecutive coronal slices in the left hemisphere (y=1335–1365), spanning the region where the ventral claustrum extends into the temporal lobe. Only every third slice (including the first and last) was manually labelled to provide input for interpolation. All methods showed more than 15% disagreement relative to manual "ground truth" (DSC<0.85), particularly struggling with the morphology of the ventral claustrum (**Fig. S6**). Additionally, disagreement between the three methods was greater than to manual ground truth (DSC=0.77-0.83). In contrast, two human raters achieve excellent agreement (DSC=0.97, see **Fig. S5**) on one representative slice, motivating our decision to proceed with full manual segmentation

Registration. Due to known subcortical alignment concerns in the original BigBrainSym dataset (24, 25), we 're-registered' BigBrain to an improved MNI-aligned BigBrain (25) using ANTs SyN, then applied this transformation to the gold standard segmentation using GenericLabel interpolation, for all spatial agreement comparisons to MRI. On the re-registered BigBrain, we also recomputed and compared all morphological metrics, but found only minute differences that did not influence the reported pattern of results; thus, to facilitate comparison to other atlasing efforts we report metrics from BigBrainSym, but make the re-registered segmentation available.

Section 3: 8-step quality control process for gold standard segmentation.

I. Raters simultaneously observed labeling in all three planes (axial, coronal, and sagittal) alongside real-time three-dimensional volumetric reconstruction in ITK-SNAP.
II. Following Kang's protocol developed for high-resolution MRI(26), raters preferentially labeled aspects of the claustrum in specific views: dorsal regions in the axial view, ventral regions in the coronal view, and the sagittal view was consulted primarily for quality control.
III. Approximately every 25mm along the anteroposterior extent, and as needed to resolve ambiguity, raters cross-referenced their label with the BigBrain dataset at 20μm in-plane resolution(27).
IV. The BigBrain dataset at 1μm in-plane resolution(27) was also cross-referenced to ensure that the claustrum label did not overlap with existing labels of nearby structures, including the putamen, amygdala, and insular cortex.
V. Upon completion of the initial segmentation, the alternate rater performed a slice-by-slice quality control review of the opposite hemisphere, correcting clear errors and resolving notable discrepancies through discussion.
VI. Within the claustrum label, voxels with intensity values more than two standard deviations below the average labeled voxel contrast were flagged. These voxels were manually reviewed by the original rater and removed as necessary to limit the erroneous inclusion of white matter and blood vessels.
VII. The claustrum label was inflated by three voxels, and voxels with intensity values greater than the average labeled voxel contrast were flagged. These voxels were manually reviewed by the original rater and included as necessary to ensure consistent gray matter inclusion along edges.
VIII. Three randomly selected coronal slices in the right hemisphere were fully and independently labeled by the alternate rater, allowing for the measurement of inter-rater agreement.

Section 4: Preprocessing and processing of MRI

Pre-processing. All participants' MP2RAGE UNI images were visually inspected for artifacts (e.g., ghosting, Gibbs ringing) and adequate subcortical contrast, and deemed suitable for inclusion. All images underwent background noise removal and bias-field correction using AFNI (28) via in-house tools (https://github.com/srikash/3dMPRAGEise), and skull-stripping using SynthStrip (29) via `mri_synthstrip` in Freesurfer v7.4.1 (30). Despite sufficient signal-to-noise in the individual runs, we constructed an unbiased average template from the three 0.5mm runs to further improve effective signal and anatomical stability, using ANTs v2.4.4 (31), with six degrees of freedom and normalised mutual information as the cost function, though the claustrum was similarly identifiable in individual runs. The 0.5mm template (averaged across three runs) and the single-scan 0.7mm and 1mm datasets were used for all subsequent analyses.

Processing. To quantify differences in claustrum visibility across the three MRI datasets, we calculated the contrast-to-noise ratio (CNR), defined as the absolute difference in mean intensity between the segmented claustrum and its surrounding white matter, normalised by the standard deviation of the white matter signal (32). In each dataset, approximately 60mm^3 of white matter voxels were selected from the left hemisphere extreme and external capsulae in the coronal view using ITK-SNAP. We also estimated intracranial volume (ICV) using the recon-all pipeline in FreeSurfer (33), for use as a covariate in sex-difference analyses.

Quality control. A second rater (NC) conducted full quality control, including manual refinements. Claustrum segmentations were verified to avoid overlap with cortical grey matter, as defined by the subject-specific cortical ribbon (ribbon.mgz) generated by the FreeSurfer recon-all pipeline (33), and with subcortical structures, specifically the putamen and amygdala, manually annotated at 0.3mm isotropic resolution on BigBrain transformed to ICBMsym space using an improved registration protocol (25).

Automated segmentation. We opted for manual segmentation after testing automated segmentation algorithms. Automation is highly desirable not only to reduce time and expertise demands, but also to curb annotation "style" that may limit cross-study comparability. Yet the same thin-sheet geometry and partial voluming that challenge humans also confound algorithms: widely used whole-brain parcellation algorithms either perform poorly (BrainSuite; Nighres), conflate the claustrum with adjacent structures (e.g., FreeSurfer SAMSEG), or omit it entirely (e.g., SPM, FSL, AFNI). Five recent bespoke algorithms have specifically targeted the claustrum, either alone or alongside a small number of other subcortical structures (4, 11–14). We applied these five algorithms to our three MRI datasets, but found that for each algorithm, in every dataset, automated segmentations were less consistent with manual segmentations than human raters were with each other, suggesting poor generalisation (**Table S14**).

Section 5: Objectives and statistical analysis.

Objective 1. Characterising claustrum morphology across resolutions.

Our first objective was to quantify how claustrum morphology varies across datasets that differ in spatial resolution and imaging modality: a high-resolution histological gold standard, its synthetically downsampled derivatives, and three rigidly-aligned *in vivo* MRI datasets. Analyses focused on eight morphometric metrics as defined above.

Analysis 1: Anatomy of the histological gold standard. First, we anatomically characterised the gold standard claustrum. Though based on a single brain, this high-resolution model preserves fine structural detail and serves as a reference for both the morphometric comparisons that follow and qualitative comparisons to prior anatomical reports.

Analysis 2: Resolution-dependent morphological degradation in downsampled gold standards. Next, we assessed how spatial resolution affects morphometric fidelity by downsampling the gold standard to a range of *in vivo* MRI-like resolutions. This simulated data, free of bias due to contrast or noise, define the theoretical maximum detail recoverable by MRI at each resolution. For each of the eight metrics (averaged across hemispheres), we fit a general linear model (GLM) with resolution and binarisation threshold as continuous fixed effects, including their interaction. Linear, quadratic, and cubic forms were tested, with the best-fitting model selected via likelihood ratio tests, Akaike Information Criterion (AIC), and Bayesian Information Criterion (BIC). Effect sizes were computed using adjusted R^2.

Analysis 3: The claustrum as captured by *in vivo* MRI. Finally, we assessed how morphometric estimates varied across the three *in vivo* MRI datasets. For each of the eight morphological metrics (averaged across hemispheres), we performed a one-way ANOVA with resolution as a fixed factor. Where significant effects were observed, pairwise comparisons were made using Tukey's HSD *post hoc* tests, and effect sizes were reported using η^2.

To evaluate measurement stability within each dataset, we quantified intra-dataset variability using the coefficient of variation (CV), defined as the ratio of the standard deviation to the mean (34). Differences in variability across datasets were assessed using Levene's test, with Games–Howell *post hoc* comparisons for pairwise differences. We expected variability to increase at lower resolutions. Finally, to assess the impact of image quality, we tested whether CNR predicted segmentation variability by regressing CNR against each participant's absolute deviation from the dataset mean (35).

Objective 2. Evaluating MRI accuracy against histological and resolution-matched gold standards.

Our second objective was to evaluate the degree of *in vivo* MRI capture by comparing segmentations to both the histological gold standard (anatomical "truth") and its synthetically downsampled derivatives at matched resolutions ('resolution ceiling'). In addition to comparing

the same eight morphometric metrics defined above, we quantified spatial correspondence using four agreement metrics on MNI-aligned MRI segmentations. The Dice Similarity Coefficient (DSC) (36) quantifies volumetric overlap, ranging from 0 (no overlap) to 1 (perfect agreement). Hausdorff Distance (HD) (37) measures the greatest distance (mm) between the closest points on each segmentation boundary, capturing maximal misalignment, ranging from 0 (perfect alignment) to infinity. Given the claustrum's high boundary-to-volume ratio (an upshot of its mediolateral thinness), and that standard spatial agreement metrics are known to penalise complex and thin structures (38), we also computed dilated DSC (dDSC), which dilates and erodes each segmentation by one voxel prior to comparison, reducing sensitivity to minor boundary mismatches (39, 40), as well as balanced average HD (baHD), which normalises directional distances based on the number of ground truth points, mitigating bias introduced by differences in segmentation size (41). Spatial agreement metrics were computed in MNI coordinates to ensure that spatial agreement reflects physical brain anatomy rather than voxel indices, enabling comparisons across datasets with different voxel resolutions.

<u>Analysis 4: Morphometric and spatial agreement between MRI and histological gold standard.</u> To assess how closely MRI segmentations approximated claustral morphology as revealed by histology, we compared the eight morphometric measurements from the three observed MRI datasets to the corresponding values derived from the histological gold standard. Deviation from the gold standard was described using percent differences and one-sample t-tests. Spatial correspondence was assessed using the four spatial agreement metrics.

<u>Analysis 5: MRI performance relative to resolution-matched gold standards.</u> To assess how closely MRI segmentations approached the theoretical limits imposed by their spatial resolution, we compared each MRI dataset to the corresponding downsampled gold standard binarised at a 50% threshold, which provides the theoretical ceiling. We chose a 50% threshold as we reasoned this is equivalent to a "majority-vote" rule, anchoring the ceiling in sampling physics rather than segmentation style. As in Analysis 4, we quantified deviation using percent differences and evaluated spatial agreement using the same four metrics. Then, to quantify how much of the theoretically achievable DSC and HD agreement MRI attained at each resolution, we calculated 'efficiency' as the ratio of MRI performance to the theoretical ceiling: (MRI dataset vs. downsampled ÷ downsampled vs. gold standard) × 100% (note that HD efficiency required an inverted calculation as lower distances indicate better performance). We are not aware of efficiency analyses for other subcortical structures or thin structures — most validations report spatial agreement metrics with histology and/or *ex vivo* MRI without accounting for resolution-imposed ceilings (24, 42) — so adopted what seemed like a fair albeit *post hoc* heuristic of ≥50-74% efficiency as adequate and ≥75-100% as high.

<u>Exploratory analyses within MRI datasets.</u>

In addition to our primary objectives of describing claustrum anatomy and characterising the capacity to image it via MRI, we conducted three exploratory investigations using MRI data to address open questions in the literature. All analyses pooled data across the three MRI datasets (n=30). For all analyses, parametric tests were applied after verifying assumptions, and multiple

comparisons were corrected using the Benjamini–Hochberg false discovery rate (FDR) (43). Spatial agreement metrics were computed in Python (v3.11.4) using scipy (44); all other statistical analyses were performed in R (v4.3.1).

Analysis 6: Inter-individual variability. To explore spatial variability in claustrum location across individuals, we created a probabilistic overlay from all MNI-aligned segmentations. Each dataset's probability volume was resampled to the highest acquired resolution (0.5 mm isotropic) using trilinear interpolation, then averaged to produce a unified probability map. Voxel values represent the proportion of participants in whom the claustrum was present at each location, providing a spatial visualisation of inter-individual boundary consistency.

Analysis 7: Hemispheric asymmetry. To assess lateral differences in claustrum morphology, we analysed left and right claustra independently using paired-samples t-tests. We computed an asymmetry index (AI) for each participant as $AI = (L - R)/(L + R)$(45), and used a GLM to test for dataset differences in AI, with 'dataset' included as a categorical covariate (0.5mm as reference).

Analysis 8: Sex differences. We analysed left and right claustra separately, assessing sex differences in each hemisphere using independent-samples t-tests. To account for known sex differences in total brain volume (46), we then performed ANCOVA including intracranial volume (ICV) included as a covariate (47).

SI REFERENCES

1. A. B. Albu, T. Beugeling, D. Laurendeau, A morphology-based approach for interslice interpolation of anatomical slices from volumetric images. *IEEE Trans. Biomed. Eng.* 55, 2022–2038 (2008).
2. A. Criminisi, Decision forests: A unified framework for classification, regression, density estimation, manifold learning and semi-supervised learning. *Found. Trends® Comput. Graph. Vis.* 7, 81–227 (2011).
3. A. Atzeni, M. Jansen, S. Ourselin, J. E. Iglesias, "A probabilistic model combining deep learning and multi-atlas segmentation for semiautomated labelling of histology" in *'Medical Image Computing and Computer Assisted Intervention-MICCAI 2018: 21st International Conference*, (Springer, 2018), pp. 219–227.
4. C. Mauri, *et al.*, A contrast-agnostic method for ultra-high resolution claustrum segmentation. *Hum. Brain Mapp.* 46, e70303 (2025).
5. B. L. Edlow, *et al.*, 7 Tesla MRI of the ex vivo human brain at 100 micron resolution. *Sci. Data* 6, 244 (2019).
6. F. Lüsebrink, A. Sciarra, H. Mattern, R. Yakupov, O. Speck, T1-weighted in vivo human whole brain MRI dataset with an ultrahigh isotropic resolution of 250 μm. *Sci. Data* 4, 170032 (2017).
7. A. Coates, N. Zaretskaya, High-resolution dataset of manual claustrum segmentation. *Data Brief* 54, 110253 (2024).
8. D. G. Cabalo, *et al.*, Multimodal precision MRI of the individual human brain at ultra-high fields. *Sci. Data* 12, 526 (2025).
9. S. Kashyap, D. Ivanov, M. Havlicek, B. A. Poser, K. Uludağ, Impact of acquisition and analysis strategies on cortical depth-dependent fMRI. *Neuroimage* 168, 332–344 (2018).
10. R. A. M. Haast, *et al.*, Insights into hippocampal perfusion using high-resolution, multi-modal 7T MRI. *Proc. Natl. Acad. Sci. U. S. A.* 121, e2310044121 (2024).
11. A. A. Albishri, S. J. H. Shah, S. S. Kang, Y. Lee, AM-UNet: automated mini 3D end-to-end U-net based network for brain claustrum segmentation. *Multimed. Tools Appl.* 81, 36171–36194 (2022).
12. S. Berman, R. Schurr, G. Atlan, A. Citri, A. A. Mezer, Automatic segmentation of the dorsal claustrum in humans using in vivo high-resolution MRI. *Cereb. Cortex Commun.* 1, tgaa062 (2020).
13. G. Brun, *et al.*, Automatic segmentation of deep grey nuclei using a high-resolution 7T magnetic resonance imaging atlas-Quantification of T1 values in healthy volunteers. *Eur. J. Neurosci.* 55, 438–460 (2022).
14. H. Li, *et al.*, Automated claustrum segmentation in human brain MRI using deep learning. *Hum. Brain Mapp.* 42, 5862–5872 (2021).
15. K. Amunts, *et al.*, BigBrain: an ultrahigh-resolution 3D human brain model. *Science* 340, 1472–1475 (2013).
16. V. S. Fonov, A. C. Evans, R. C. McKinstry, C. R. Almli, D. L. Collins, Unbiased nonlinear average age-appropriate brain templates from birth to adulthood. *Neuroimage* 47, S102 (2009).
17. H. Brockhaus, Die Cyto-und Myeloarchitektonik des Cortex claustralis und des Claustrum beim Menschen. *J Psychol Neurol* 49, 249–348 (1940).
18. A. S. Rae, The form and structure of the human claustrum. *J. Comp. Neurol.* 100, 15–39 (1954).
19. I. N. Filimonoff, The claustrum, its origin and development. *J. Hirnforsch.* 8, 503–528 (1966).
20. R. Druga, "The structure and connections of the claustrum" in *The Claustrum*, (Elsevier, 2014), pp. 29–84.

21. S.-L. Ding, *et al.*, Comprehensive cellular-resolution atlas of the adult human brain. *J. Comp. Neurol.* 524, 3127–3481 (2016).
22. J. K. Mai, M. Majtanik, G. Paxinos, *Atlas of the Human Brain* (Academic Press, 2015).
23. J. B. Smith, *et al.*, The relationship between the claustrum and endopiriform nucleus: A perspective towards consensus on cross-species homology. *J. Comp. Neurol.* 527, 476–499 (2019).
24. K. R. Sitek, *et al.*, Mapping the human subcortical auditory system using histology, postmortem MRI and in vivo MRI at 7T. *Elife* 8 (2019).
25. Y. Xiao, *et al.*, An accurate registration of the BigBrain dataset with the MNI PD25 and ICBM152 atlases. *Sci Data* 6, 210 (2019).
26. S. S.-S. Kang, J. Bodenheimer, K. Morris, T. Butler, A comprehensive and reliable protocol for manual segmentation of the human claustrum using high-resolution MRI. *Brain Struct. Funct.* 230, 134 (2025).
27. K. Amunts, H. Mohlberg, S. Bludau, K. Zilles, Julich-Brain: A 3D probabilistic atlas of the human brain's cytoarchitecture. *Science* 369, 988–992 (2020).
28. R. W. Cox, AFNI: Software for Analysis and Visualization of Functional Magnetic Resonance Neuroimages. *Comput. Biomed. Res.* 29, 162–173 (1996).
29. A. Hoopes, J. S. Mora, A. V. Dalca, B. Fischl, M. Hoffmann, SynthStrip: skull-stripping for any brain image. *Neuroimage* 260, 119474 (2022).
30. B. Fischl, FreeSurfer. *Neuroimage* 62, 774–781 (2012).
31. N. J. Tustison, *et al.*, The ANTsX ecosystem for quantitative biological and medical imaging. *Sci. Rep.* 11, 9068 (2021).
32. S. D. Wolff, R. S. Balaban, Assessing contrast on MR images. *Radiology* 202, 25–29 (1997).
33. O. Tange, *GNU Parallel 20240622 ('34 counts')* (Zenodo, 2024).
34. R. R. Sokal, F. J. Rohlf, *Biometry*, 3rd Ed. (W.H. Freeman, 1995).
35. J. S. Wonderlick, *et al.*, Reliability of MRI-derived cortical and subcortical morphometric measures: effects of pulse sequence, voxel geometry, and parallel imaging. *Neuroimage* 44, 1324–1333 (2009).
36. L. R. Dice, Measures of the Amount of Ecologic Association Between Species. *Ecology* 26, 297–302 (1945).
37. D. P. Huttenlocher, G. A. Klanderman, W. J. Rucklidge, Comparing images using the Hausdorff distance. *IEEE Trans. Pattern Anal. Mach. Intell.* 15, 850–863 (1993).
38. L. Maier-Hein, *et al.*, Metrics reloaded: recommendations for image analysis validation. *Nat. Methods* 21, 195–212 (2024).
39. A. C. Trutti, *et al.*, A probabilistic atlas of the human ventral tegmental area (VTA) based on 7 Tesla MRI data. *Brain Struct. Funct.* 226, 1155–1167 (2021).
40. P.-L. Bazin, A. Alkemade, M. J. Mulder, A. G. Henry, B. U. Forstmann, Multi-contrast anatomical subcortical structures parcellation. *Elife* 9 (2020).
41. O. U. Aydin, *et al.*, On the usage of average Hausdorff distance for segmentation performance assessment: hidden error when used for ranking. *Eur Radiol Exp* 5, 4 (2021).
42. J. E. Iglesias, *et al.*, A probabilistic atlas of the human thalamic nuclei combining ex vivo MRI and histology. *Neuroimage* 183, 314–326 (2018).
43. Y. Benjamini, Y. Hochberg, Controlling the false discovery rate: A practical and powerful approach to multiple testing. *J. R. Stat. Soc. Series B Stat. Methodol.* 57, 289–300 (1995).
44. P. Virtanen, *et al.*, SciPy 1.0: fundamental algorithms for scientific computing in Python. *Nat. Methods* 17, 261–272 (2020).
45. A. W. Toga, P. M. Thompson, Mapping brain asymmetry. *Nat. Rev. Neurosci.* 4, 37–48 (2003).
46. A. N. V. Ruigrok, *et al.*, A meta-analysis of sex differences in human brain structure. *Neurosci. Biobehav. Rev.* 39, 34–50 (2014).

47. C. W. S. Pintzka, T. I. Hansen, H. R. Evensmoen, A. K. Håberg, Marked effects of intracranial volume correction methods on sex differences in neuroanatomical structures: a HUNT MRI study. *Front. Neurosci.* 9, 238 (2015).
